# Metabolism, pharmacokinetics, and bioavailability of cannabigerol in horses following intravenous and oral administration with micellar and oil formulations

**DOI:** 10.3389/fvets.2025.1688214

**Published:** 2025-10-29

**Authors:** Juan Manuel Serrano-Rodríguez, Raquel Miraz, Aritz Saitua, Elisa Díez de Castro, Carlos Ledesma-Escobar, Carlos Ferreiro-Vera, Feliciano Priego-Capote, Verónica Sánchez de Medina, Antonia Sánchez de Medina

**Affiliations:** ^1^Pharmacology Area, Department of Nursing, Pharmacology and Physiotherapy, Veterinary Faculty, University of Córdoba, Córdoba, Spain; ^2^Veterinary Clinical Hospital, University of Cordoba, Córdoba, Spain; ^3^Department of Animal Medicine and Surgery, Veterinary Faculty, University of Cordoba, Córdoba, Spain; ^4^Equine Sports Medicine Center CEMEDE, Department of Animal Medicine and Surgery, Veterinary Faculty, University of Córdoba, Córdoba, Spain; ^5^Department of Analytical Chemistry, Science Faculty, University of Córdoba, Córdoba, Spain; ^6^Chemical Institute for Energy and Environment (IQUEMA), Campus of Rabanales, University of Córdoba, Córdoba, Spain; ^7^Phytoplant Research S.L.U., Córdoba, Spain

**Keywords:** metabolism, pharmacokinetics, bioavailability, cannabigerol, horses, micellar, oil

## Abstract

**Introduction:**

Cannabigerol (CBG) is a non-psychoactive cannabinoid with growing interest in veterinary medicine; however, its pharmacokinetic profile in horses remains unknown. Understanding its absorption, distribution, metabolism, and elimination is essential to optimizing dosing strategies and evaluating its potential for clinical use in equine patients.

**Methods:**

A prospective crossover study was conducted in eight healthy adult horses to assess the *in vivo* metabolism and the pharmacokinetics after intravenous (IV) administration at 1 mg/kg and oral administrations at 10 mg/kg with two formulations (micellar and oil). Plasma concentrations of CBG and its main metabolite, CBG-glucuronide (CBG-G), were analyzed by LC-MSMS and modeled using a non-linear mixed effects model with MonolixSuite®. The model estimated the bioavailability, metabolic conversion, and absorption parameters. Furthermore, Monte Carlo simulations were performed to predict and evaluate the drug exposure after a multiple-dose regimen.

**Results:**

High *in vivo* metabolism was observed with the formation of epoxy and hydroxy metabolites via phase I reactions, and CBG-G was the main metabolite from phase II reactions (75% of biotransformation). After IV administration, CBG showed a high volume of distribution (V_ss_ = 74 L/kg) and systemic clearance (Cl = 1.67 L/h/kg), with a terminal half-life of approximately 29 h. The oral bioavailability was estimated at 28% between formulations, and an extensive presystemic metabolism was obtained with metabolite/parent AUC ratios exceeding 50. The micellar formulation showed a shorter time to achieve maximum concentration (T_max_) and faster absorption as compared to the oil formulation. The Monte Carlo simulations of multiple oral doses (10 mg/kg q24 h for 14 days) predicted differences between formulations. No adverse clinical effects were observed during the study.

**Discussion:**

This study shows the first evaluation of the *in vivo* metabolism and pharmacokinetics of CBG in horses after IV and oral administration. The findings highlight extensive metabolite formation with significant glucuronidation, a large distribution volume, and high clearance. While both oral formulations produced similar systemic exposure, the faster absorption with the micellar formulation may inform clinical decisions depending on therapeutic goals. These data support the potential use of CBG in horses and offer a foundation for further studies in equine medicine.

## Introduction

Cannabigerol (CBG) is a non-psychoactive cannabinoid derived from the plant *Cannabis sativa L.,* recently recognized for its potential therapeutic applications (1). Unlike other related drugs such as cannabidiol (CBD), which has been extensively studied in recent years, the research on CBG remains limited. Despite this, the preliminary evidence indicates that CBG may have significant pharmacological properties, warranting further research in human or veterinary medicine (2).

CBG interacts with the endocannabinoid system, particularly targeting the cannabinoid receptors CB1 and CB2 as a weak and partial agonist, respectively (3). These receptors are distributed across the central nervous system (CNS), peripheral tissues, and immune system, with CB1 predominantly located in the brain and peripheral nerves, influencing neural activity and pain perception, among others, and CB2 primarily associated with the immune system, modulating inflammation and immune responses (4). Additionally, CBG exhibits various *in vitro* and *in vivo* effects that can be explained by the action with other targets: transient receptor potential (TRP) channels (vanilloid receptors TRPV), G protein-coupled receptors (α_2_ adrenergic or the serotonin receptor 5-HT_1A_), and antimicrobial activity against *Streptococcus mutants* (5, 6). Thus, CBG is an interesting cannabinoid for clinical research and applications, including anti-inflammatory, neuroprotective, analgesic, anxiolytic, and antibacterial properties (7–9).

From a pharmacokinetics (PK) point of view, publications describing the plasma disposition of CBG after different doses or routes remain limited in comparison to CBD. In fact, to the best knowledge of the authors, only limited studies by oral and intraperitoneal routes in rodents, humans, and dogs have been described, but the intravenous (IV) administration and the oral bioavailability are unknown (10–12). In horses, a recent study reported pain reduction in animals with osteoarthritis (OA) using oral plant extracts with CBG, but without concentration analysis (13). The PK of CBG in horses has not been described, and this knowledge is essential to establish dosing and clinical use recommendations. Nevertheless, CBG is a very lipophilic drug (LogP = 7.04) that undergoes intense hepatic metabolism with the formation of epoxides and hydroxides from phase I reactions and glucuronides from phase II reactions in humans and small animals (14, 15). However, the biotransformation of CBG and the resultant metabolite levels have not been described in horses. Moreover, it is known that the hepatic first-pass effect limits the oral bioavailability of drugs in horses (16–18). Consequently, the development and evaluation of different oral formulations should be determined (19–21). Subsequently, the study and description of the PK of CBG could improve the knowledge of this drug in horses, following the main recommendations on these studies in equines (22).

The present study has been designed to first, detect and evaluate the *in vivo* metabolic pathways of CBG in healthy horses (*n* = 8); second, describe the PK properties following IV and oral administration of CBG; third, study and compare the oral absorption and bioavailability of CBG with two formulations; and fourth, simulate and evaluate different treatment regimens after multiple doses with the Monte Carlo simulation.

## Materials and methods

### Experimental design and animals

The number of horses was calculated following the indications described for a rich sampling design in PK studies using the Cl as the main parameter (23, 24). However, no previous references about IV dosing of CBG were found, and it was assumed to have a low-moderate variability close to 50% in the coefficient of variation (%CV) to the estimated Cl (25). Finally, several animals greater than or equal to eight produced a power greater than 90% with a standard deviation of logCl of 0.31. The calculation was developed in RStudio software (R 4.3.3, R Development Core Team, R Foundation for Statistical Computing, Vienna, Austria) (23).

For the study, eight healthy horses (four non-pregnant mares and four geldings) of different breeds (six Andalusian horses and two cross breed), weighing 451 ± 49.4 kg and aged 16 ± 3.7 years, were included. The animals were housed separately in stable boxes with the same straw bedding material. They were fed with good-quality hay twice a day and had free access to water. They were considered healthy based on physical examination, clinical history, and standard hematological and biochemical analyses. Additionally, none of the animals had received any treatments for at least 1 month before the study.

### Drug formulation preparation, administration, and blood sampling

Cannabigerol (CBG), a pure substance (≥99% purity), sourced from the registered medicinal *Cannabis sativa* L. variety Octavia (Community Plant Variety Office registration number: 20170148, Phytoplant Research S.L.U.), was employed in all assays detailed herein. The experimental formulations were prepared 24 hours before administration. For the IV study, CBG was dissolved in Chremophor at 5% (Kolliphor® RH 40, Sigma–Aldrich, Merck Life Science S.L.U.), ethanol at 5% (anhydrous, denatured, Sigma–Aldrich, Merck Life Science S.L.U.), and saline solution at 0.9% (Fisiovet®, Braun VetCare, S.A.). A final concentration of CBG at 10 mg/mL was obtained, mixed, filtered, and stored at 4 °C before trials. For oral administration, an oil-based formulation was prepared by dissolving the necessary amount of CBG in sesame oil (Sigma-Aldrich, Merck Life Science S.L.U.) to yield a final concentration of 200 mg/mL. Similarly, a micellar formulation of CBG was prepared at the same concentration. A formulation of 200 mg/L of CBG was obtained, and the critical micellar concentration (CMC) of the surfactant required for micelle formation was 2.8 μmol/L. The particle size was 39.8 nm using an analogous dissolution process (16). Both oral formulations were stored at 4 °C before experimental use.

Drug administration in this research was conducted in three different treatments. In the first treatment, each horse received CBG at a dose of 1.0 mg/kg by IV route over a period of 5 min. After 2 weeks of washout, the second and third treatments were undertaken, using a blinded and randomized Latin square (2 × 2) design using CBG in sesame oil formulation and in micellar formulation at a 10 mg/kg dose by syringe, with 2 weeks of washout between treatments. Food was withdrawn from 12 h before administration of the drug up to 8 h after.

Catheters in the jugular vein were placed aseptically on the same day of each of the three trials. Blood samples (5 mL) were obtained at the following times: 0 (pre-dosing), 5, 10, 15, 30, 45 min, and 1, 2, 4, 6, 8, 12, 24, 36, 48, and 72 h in lithium heparin tubes. The samples were immediately centrifuged at 1500 g for 15 min. The plasma was separated and stored frozen (−80 °C). Furthermore, 2 mL blood samples were collected into EDTA tubes for hematological analysis, and an equal volume was obtained into lithium heparin tubes for biochemical assessment. Sampling was performed before drug administration and at 72 h post-dosing.

### Drug analysis and metabolite determination

Plasma concentrations of CBG, as well as the identification and detection of metabolites, were measured within 4 months after freezing using a validated liquid chromatography–tandem mass spectrometry (LC–MS/MS) method (26).

An aliquot of plasma (400 μL) was mixed with 1,200 μL of methanol for protein precipitation, and 50 μL of hydroxytyrosol (1 mg/L) was used as an internal standard (Sigma–Aldrich, Merck Life Science S.L.U.). The mixture was shaken in the vortex for 5 min and centrifuged at 4 °C and 14,000 rpm for 15 min. The centrifugation was transferred to a new tube and evaporated under vacuum with centrifugation until dryness. Finally, the residue was redissolved in 50 μL of methanol and transferred into an amber glass vial for analysis by LC–MS/MS.

Samples were analyzed using LC–MS/MS in high-resolution mode with data-independent acquisition (DIA) mode using an LC-QTOF MS/MS system. Chromatographic separation was performed on a 1,200 Series Agilent (Palo Alto, CA, USA) LC system equipped with a C_18_ reversed-phase column (Zorbax Eclipse Plus C18 HD 3.0 × 100 mm, 1.8 μm) and a guard column (Zorbax Eclipse Plus C_18_ HD 3.0 × 5 mm, 1.8 μm), using water (phase A) and acetonitrile (phase B) as mobile phases, both with 0.1% of formic acid as an ionization agent. The following elution gradient was used: from 0 to 0.5 min, 70% phase B; from 0.5 to 7 min, a linear increase from 70 to 100% phase B; from 7 to 15 min, maintained at 100% phase B to ensure the elution of all sample components. The column was then equilibrated to initial conditions for 5 min before the following analysis. The chromatographic flow rate was 0.5 mL/min, and the injection volume was 10 μL. The LC system was coupled to a 6,540 quadrupole-time-of-flight detector (QTOF MS/MS; Agilent Technologies, Santa Clara, CA, USA). Electrospray ionization (ESI) parameters in both negative and positive modes were as follows: nebulizer gas, 40 psi; flow rate and temperature of drying gas (N_2_), 12 L/min and 325 °C, respectively; capillary voltage, ±3.5 kV; Q1, skimmer, and octapole voltages, 130, 65, and 750 V, respectively.

DIA parameters were set as follows: the acquisition rate was 5 spectra/s with three channels at variable collision energies (CE, 0, 20, and 40 eV), and a cycle time of 1 s per channel (total cycle time 3 s). The acquisition range was 40–1,100 m/z for all channels. The measurement of accurate m/z values on the 0 eV channel (without fragmentation) in all analyses was ensured through continuous internal calibration using the signals at m/z 112.9856 (trifluoroacetic acid anion) and 1033.9881 (HP-921, hexakis(1H,1H,3H-tetrafluoropropoxy) phosphazine).

MassHunter Qualitative Analysis software (version B7.00; Agilent Technologies, Santa Clara, CA, USA) was used to integrate the signals obtained using LC–MS/MS and to construct the data matrix. Metabolite annotation was also performed using MetaboMSDIA (Version 1.00) and the R software package (26), which allows the obtainment of multiplexed MS2 spectra for CBG, and tentative identification of CBG-metabolites was based on finding out the characteristic fragmentation of CBG and neutral mass losses such as the glucuronide (m/z 176.0324). Briefly, the software extracts all signals in the 0 eV channel, which are considered precursor ions, as well as those in the 20 and 40 eV channels, which contain signals for product ions. Then, considering parameters such as peak shape, intensity, and retention time tolerance, the application associates the precursor ions with their corresponding product ions to obtain an MS2 spectrum for each precursor ion at the different collision energies applied. All experimental MS2 spectra obtained were converted into CEF archives for comparison with CBG MS2 spectral information using PCDL Manager software (version B7.00; Agilent Technologies, Santa Clara, CA, USA).

Before quantification and after preliminary screening, CBG and different metabolites were identified. From phase I reactions, 8′-hydroxy-CBG, reduced 8′-hydroxy-CBG, epoxide-CBG (sum of isomers), dihydroxy-CBG (sum of isomers), and reduced dihydroxy-CBG (sum of isomers) were detected. From phase II reactions, only CBG-glucuronide (CBG-G) was identified. However, due to the lack of analytical standards for phase I metabolites, only the glucuronide was quantified (Cannabigerol-O-beta-D-glucuronide sodium salt solution, Sigma–Aldrich, Merck Life Science S.L.U.). Consequently, CBG, as a parent drug and CBG-G as the main metabolite were included in the subsequent quantitative analysis.

A calibration model for CBG and CBG-G in the 1–2,000 ng/mL concentration range was performed. For those metabolites without available standards, relative quantitation was calculated as a CBG equivalent. Quality controls were performed to determine the precision and accuracy using blank equine plasma spiked with concentrations of CBG, CBG-G, and the internal standard. Intra-day precision was obtained from nine replicates of three calibration standards, yielding a relative standard deviation (RSD) below 3.5%. Inter-day precision was evaluated from plasma analyses conducted across three different days, with RSD values below 4.1%. The recovery obtained fell in the range of 96.7 ± 0.02% to 98.2 ± 0.07%. After analysis, the limits of detection (LOD) were 0.0001 and 0.0003 nmol/L for CBG and CBG-G, and the limits of quantification (LOQ) were 0.0004 and 0.0008 nmol/L, respectively. The concentrations of CBG and CBG-G determined in this way were used for the PK modeling in the next stage of the study.

### Pharmacokinetic modeling, Monte Carlo simulations, and statistical analysis

The plasma concentrations over time of CBG and CBG-G were simultaneously modeled with a non-linear mixed effect approach (NLME) using Monolix 2024R1® Suite software (Simulations Plus/Lixoft, Ltd., Lancaster, CA, US).

In the first step, different structural models were evaluated, including one, two, or three compartments, with single or multiple absorption rates with delayed absorption, transit compartments, or time-dependent absorption equations. In a second step, the selection of the final model was done following a reduction of the variability and according with different statistical criteria such as the likelihood ratio tests: −2·log-likelihood (−2xLL), the Akaike information criterion (AIC) and the Bayesian information criterion (BIC) (24). Moreover, the goodness of fit plots was also checked in each model with visual inspection of the scatter plots of population/individual predicted versus observed concentrations, the population/individual-weighted residuals (PWRES and IWRES) versus predictions/time and finally and the visual predictive check plots (VPC) (27).

The final PK model was built using two compartments for the parent drug and one compartment for the metabolite, with time-dependent absorption for the oral route and linear elimination for both drug and metabolite molecules. The model was written as an ordinary differential equations system from Equation 1 to Equation 4. The oral absorption was modeled using a Weibull function and included into the model and showed in Equation 5.


(1) $$\frac{dA_{1}}{\mathrm{dt}}=F\cdot k_{\mathrm{aW}}\cdot A_{4}-\frac{\mathrm{Cl}}{V_{c}}\cdot A_{1}-\frac{Q}{V_{c}}\cdot A_{1}+\frac{Q}{V_{p}}\cdot A_{2}$$

(2) $$\frac{dA_{2}}{\mathrm{dt}}=\frac{Q}{V_{c}}\cdot A_{1}-\frac{Q}{V_{p}}\cdot A_{2}$$

(3) $$\frac{dA_{3}}{\mathrm{dt}}=\frac{\mathrm{Cl}}{V_{c}}\cdot F_{m}\cdot A_{1}-\frac{{\mathrm{Cl}}_{m}}{V_{m}}\cdot A_{3}$$

(4) $$\frac{dA_{4}}{\mathrm{dt}}=-F\cdot k_{\mathrm{aW}}\cdot A_{4}$$

(5) $$k_{\mathrm{aW}}=(k_{a}\cdot \beta )\cdot {\left(k_{a}\cdot t\right)}^{\beta -1}$$

Where A_1_, A_2_ and A_4_ are the amounts of CBG in central, peripheral and depot compartments, respectively; A_3_ is the amounts of CBG-G in the metabolite compartment. Concentrations of CBG and CBG-G were calculated as C_1_ = A_1_/V_c_ and C_3_ = A_3_/V_m_, respectively.

The parameters determined by the model from CBG included: k_a_, the absorption rate constant; β, the shape parameter that influences the slope of the absorption phase; F, the oral bioavailability; V_c_, the volume of distribution at the central compartment; Cl, the clearance; Q, the intercompartmental clearance; V_p_, the volume of distribution at the peripheral compartment. For the metabolite CBG-G, the following were: F_m_, the fraction of CBG converted to CBG-G; Cl_m_, the clearance; V_m_, the volume of distribution at the metabolite compartment.

Each parameter determined by the model for each animal was described in the following form (Equation 6):


(6) $$\theta_{i}=\theta_{\text{typical}}\cdot e^{\eta_{\mathrm{IIV}}}\cdot e^{\eta_{\mathrm{IOV}}}$$

Where θ_i_ is the parameter estimate for each i_th_ animal from the set of i = n animals (*n* = 8); θ_typical_ is the typical value estimated by the model; the parameter η_IIV_ represents the inter-individual variability (IIV) associated with each i_th_ animal from the corresponding typical value θ_typical_; η_IOV_ is the inter-occasion variability (IOV) associated with each i_th_ animal from each assay evaluated in consonance with the θ_typical_ value. A log-normal distribution was assumed for all parameters of the model, whereas that the oral bioavailability was assumed a logit-normal distribution to bound predictions between 0 and 1 (24). Moreover, after previous analysis of our data with the model, it was assumed that 75% of CBG was eliminated via metabolic conversion to CBG-G, and the fraction of CBG metabolized to CBG-G was fixed to 0.75 (15). Subsequently, two covariates were studied to evaluate their influence on the disposition of CBG and CBG-G: the weight of animals as a continuous covariate and the type of formulation as a categorical covariate (coded A for micellar and B for oil formulation). Firstly, we tested with the method of conditional sampling for a stepwise approach based on correlation tests (28). Secondly, their effects were studied using the Pearson correlation test, the Wald test, and the analysis of variance (ANOVA). The covariates were included if they showed statistical significance (*p* < 0.05) and reduced the IIV, IOV, −2xLL, AIC, and BIC values, respectively. The covariates studied were retained in the final model if they produced a ≥ 10 reduction in BIC criteria (24, 28). After this analysis, only the effect of the oral formulation was included as a categorical covariate, and Equation 6 was modified to produce Equation 7 with Cov_θ(i)_ as the exponent for the covariate effect:


(7) $$\theta_{i}=\theta_{\text{typical}}\cdot e^{\eta_{\mathrm{IIV}}}\cdot e^{\eta_{\mathrm{IOV}}}\cdot e^{{\mathrm{cov}}_{\mathrm{\theta i}}}$$

The robustness of the PK model was evaluated using a convergence assessment with 500 replicates, the shrinkage values for each estimated parameter, and a non-parametric bootstrap analysis with a 95% confidence interval (29).

The residual variability from the predicted concentrations was studied, evaluating the error models (additive, proportional, or combined). At last, a proportional error model was selected and described as C_obs_ = C_pred_ + b C_pred_ *ε*. Where C_obs_ is the observed concentration, C_pred_ is the predicted concentration, and b is the component for the residual error (24).

Other PK parameters obtained for the drug and metabolite were the area under the curve (AUC), the area under the curve from zero to 24 h (AUC_24_), the volume of distribution at steady state V_ss_, the elimination half-lives (t_1/2_), the maximum concentrations observed (C_max_), the time to reach C_max_ (T_max_), and the ratio of metabolite to the parent drug AUC_m_/AUC_p_ (30, 31).

The final PK model obtained was imported to Simulx, the simulation package included in the MonolixSuite®, and the individual predictions were used to carry out the Monte Carlo simulations. Firstly, oral simulated dose regimens of CBG were built in a hypothetical scenario of multiple doses at 10 mg/kg every 24 h for 14 days of treatment for each formulation (*n* = 5,000 per group). Secondly, AUC_24_ and AUC values at steady state were obtained and used to calculate the simulated accumulation index and the C_max_ and T_max_ values at these times.

The analysis and comparison of parameters were conducted using descriptive statistics and distribution tests using RStudio software. The data were normally distributed, and parametric tests were used. The ANOVA test was used to compare differences between parameters. When significant differences were found, a t-test was used as a second post-hoc test (pairwise comparison). A Bonferroni correction was applied for this t-test. The significance level was set at *p* < 0.05.

## Results

Clinical examination of all animals after each treatment showed no abnormalities. Biochemical and hematology values were within the range of variation established in our hospital for horses. The treatments were well tolerated by the animals, and the oral doses were well taken. Consequently, no local or systemic adverse reactions were observed in this research (Supplementary Table S4).

**Figure 1:**
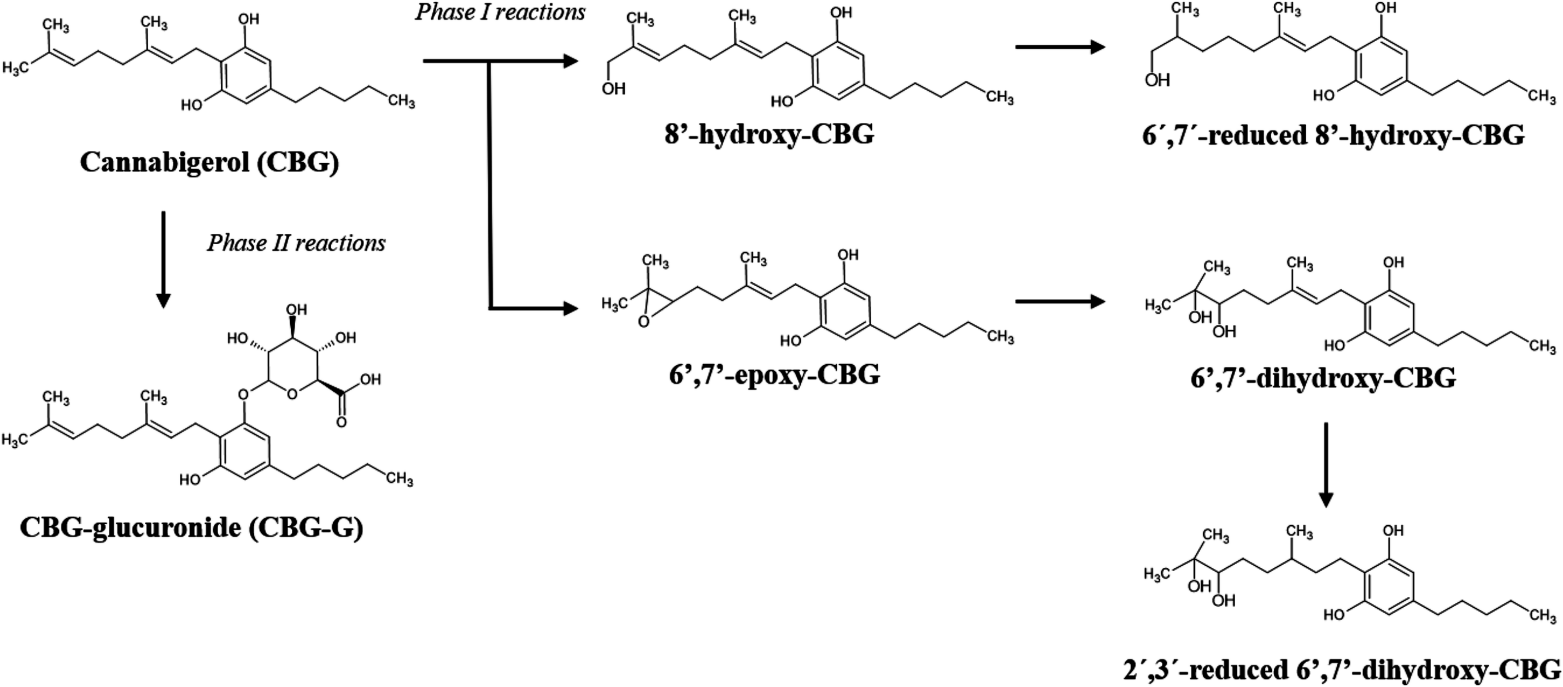
Proposed pathway of phase I and phase II metabolic reactions of CBG following intravenous (IV) and oral administration in eight healthy adult horses (*n* = 8). For phase I reactions, the most abundant oxidative and hydroxylated metabolites are shown. For phase II reactions, the major conjugated metabolite, cannabigerol glucuronide (CBG-G), was detected. Identification was based on liquid chromatography–tandem mass spectrometry (LC–MS/MS). CBG, cannabigerol; CBG-G, cannabigerol glucuronide.

Based on LC–MS/MS data obtained, different metabolites of CBG were identified, supporting the existence of two major pathways for phase I and one glucuronidation pathway for phase II. The detected phase I metabolites included hydroxylated, reduced, and epoxidized derivatives. The glucuronide conjugate (CBG-G) was also observed in the phase II reaction. Figure 1 shows the main proposed metabolic pathways, and Supplementary Figures S1–S6 include additional isomers, pathways, chromatograms, and MS/MS spectra.

**Figure 2:**
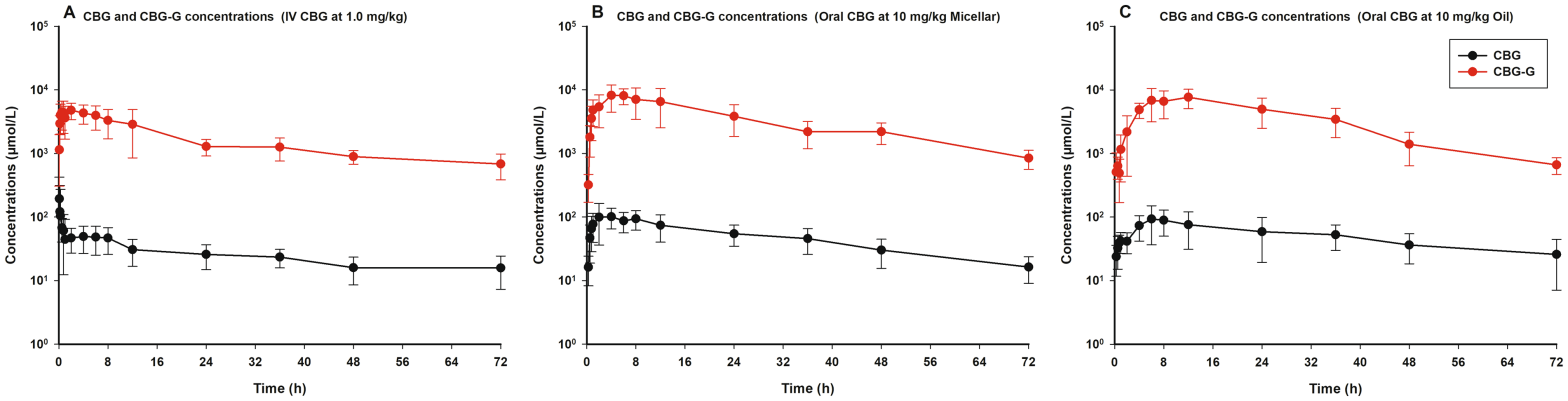
Observed plasma concentrations of cannabigerol (CBG) and its main phase II metabolite cannabigerol glucuronide (CBG-G) in horses following three different formulations. Mean plasma concentrations ± standard deviation (SD) is presented for CBG (black lines) and CBG-G (red lines) in eight healthy adult horses (*n* = 8). **(A)** Left panel: intravenous (IV) administration of CBG at 1.0 mg/kg. **(B)** Central panel: oral micellar administration of CBG at 10 mg/kg. **(C)** Right panel: oral oil administration of CBG at 10 mg/kg.

**Figure 3:**
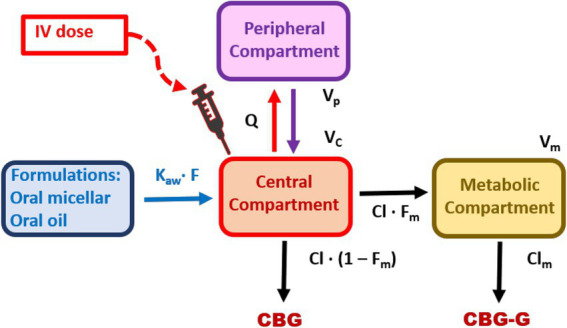
Diagram of the parent-metabolite model used to describe the simultaneous disposition of cannabigerol (CBG) and its glucuronidated metabolite (CBG-G) in horses. This model was used to characterize the plasma concentration–time profiles of CBG and CBG-G following intravenous (IV) administration (1.0 mg/kg) and oral administration (10 mg/kg) of CBG using micellar and oil-based formulations, respectively. Model parameters include, F, oral bioavailability; ka, absorption rate constant; *β*, slope of the absorption phase (oral routes); Vc, volume of distribution in the central compartment; Cl, systemic clearance; Q, intercompartmental clearance; Vp, volume of distribution in the peripheral compartment; Fm, fraction of CBG converted to CBG-G; Clm, clearance of CBG-G; Vm, volume of distribution of CBG-G.

The observed plasma concentrations of CBG and CBG-G after IV administration of CBG at 1.0 mg/kg and oral administration at 10 mg/kg by micellar and oil formulations are shown in Figure 2, with black lines for CBG and red lines for CBG-G, respectively. A schematic diagram of the PK model is presented in Figure 3 and the code is described in the Supplementary material.

**Figure 4:**
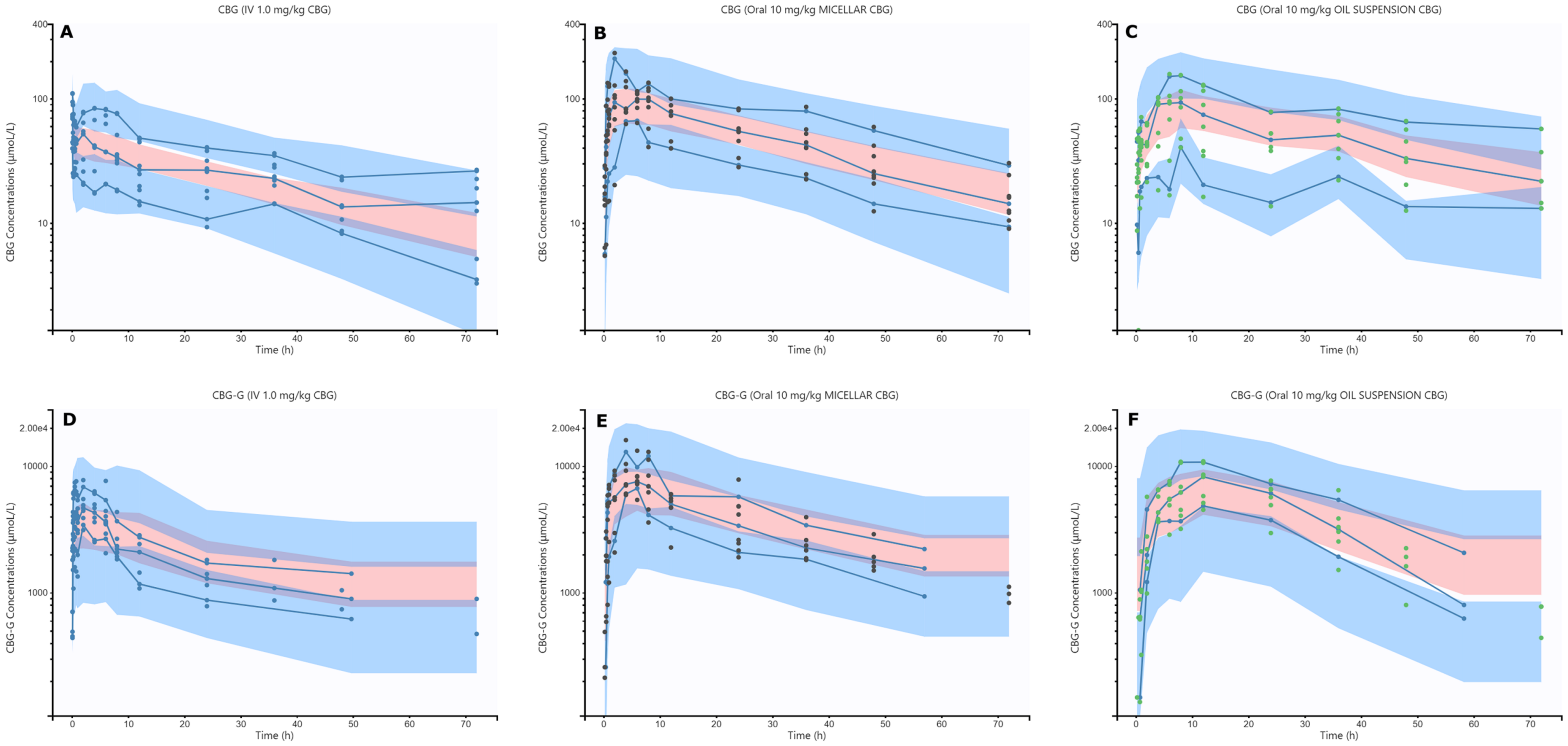
Visual predictive check (VPC) plots for cannabigerol (CBG) and its glucuronidated metabolite (CBG-G), stratified by route of administration in horses. **(A)**: CBG plasma concentrations after intravenous (IV) administration of 1.0 mg/kg CBG. **(B)**: CBG plasma concentrations after oral micellar administration of 10 mg/kg CBG. **(C)**: CBG plasma concentrations after oral oil administration of 10 mg/kg CBG. **(D)**: CBG-G plasma concentrations after IV administration of 1.0 mg/kg CBG. **(E)**: CBG-G plasma concentrations after oral micellar administration of 10 mg/kg CBG. **(F)**: CBG-G plasma concentrations after oral oil administration of 10 mg/kg CBG. The 10th and 90th percentiles of the interval prediction are shown in blue, and the predicted 50th percentiles (median) are shown in pink. Empirical percentiles of the observed data (10th, 50th, and 90th) are displayed as blue lines. Observed concentrations of CBG and CBG-G are displayed in blue, black, and green for IV, micellar, and oil administration, respectively.

The main parameters obtained by the PK model are shown in Table 1, and the secondary in Table 2. The plasma concentrations of CBG and CBG-G were accurately described by the model, with random effects ranging from 14 to 54% and shrinkage values between −14.4 and 6.25% (Supplementary Table S1) (32). The visual predictive check (VPC) plots for both compounds (Figure 4) showed most values within the 90% prediction intervals and centered around the median (50th of the percentile) (33). Additional diagnostic tools, such as observation versus prediction plots, residual plots, and non-parametric bootstrap analysis, supported the robustness of the model developed (Supplementary Table S1; Supplementary Figures S7–S10).

**Table 1:** Pharmacokinetic parameters of CBG and CBG-G in horses after intravenous CBG at 1.0 mg/kg and oral CBG at 10 mg/kg by micellar and oil formulations, respectively.

Parameter	Estimate	IIV (%)	IOV (%)	Parameter by category	Estimate
F	0.28	25.52	13.93	–	–
k_a_ (1/h)	0.99	46.64	37.99	k_a_micellar formulation_ (1/h)	0.99
β	1.59	26.01	12.8	β__micellar formulation_	1.59
Cl (l/h/kg)	1.67	27.95	–	k_a_oil formulation_ (1/h)	0.31
V_c_ (L/kg)	32.15	66.74	–	β__oil formulation_	0.70
Q (L/h/kg)	154.5	–	–	–	–
V_p_ (L/kg)	36.12	21.22	–	–	–
V_m_ (L/kg)	0.0047	53.58	–	Residual error	–
F_m_	0.75	–	–	b_1_	0.29
Cl_m_ (L/h/kg)	0.016	32.23	–	b_2_	0.41

F, bioavailability after oral administration; k_a_, absorption rate constant; β, slope of the absorption phase; V_c_, volume of distribution at central compartment; Cl, clearance; Q, intercompartmental clearance; V_p_, volume of distribution at peripheral compartment; F_m_, fraction of CBG converted to CBG-G; Cl_m_, clearance of CBG-G; V_m_, volume of distribution of CBG-G. IIV and IOV, interindividual and interoccasion variabilities expressed as percentage of coefficient of variation. Cov_ka_formulation_, categorical covariate for the absorption rate constant; Cov_β_formulation_, categorical covariate for slope of the absorption phase. b_1_ and b_2_, residual errors for CBG and CBG-G, respectively.

**Table 2:** Secondary pharmacokinetic parameters for CBG and CBG-G after intravenous CBG at 1.0 mg/kg and oral administration of 10 mg/kg of CBG by oil or micellar formulations, respectively.

IV formulation of CBG at 1.0 mg/kg				
Parameter	CBG		CBG-G	
	Estimate	% CV	Estimate	% CV
AUC_24_ (μmol/L·h)	855.75	41.45	62419.46	25.49
AUC (μmol/L·h)	2376.23	46.82	105071.97	42.44
C_max_ (μmol/L)	–	–	5508.28	31.05
T_max_ (h)	–	–	2.28	81.47
t_1/2_ (h)	29.22	62.89	21.01	69.23
V_ss_ (L/kg)	74.31	27.69	–	–
AUC_m_/AUC_p_	–	–	54.25	58.26

Oral micellar formulation at 10 mg/kg				
Parameter	CBG		CBG-G	
	Estimate	% CV	Estimate	% CV
AUC_24_ (μmol/L·h)	1817.02	27.15	138645.04	33.21
AUC (μmol/L·h)	4109.79	31.02	247414.22	26.80
C_max_ (μmol/L)	132.70	36.66	10400.83	29.24
T_max_ (h)	4.00	46.29	5.75	29.03
t_1/2_ (h)	28.58	30.39	23.56	66.53
AUC_m_/AUC_p_	–	–	64.41	35.56

Oral oil formulation at 10 mg/kg				
Parameter	CBG		CBG-G	
	Estimate	% CV	Estimate	% CV
AUC_24_ (μmol/L·h)	1635.11	48.24	133925.40	31.00
AUC (μmol/L·h)	5136.01	43.87	306448.70	33.14
C_max_ (μmol/L)	101.38	46.55	8003.28	33.24
T_max_ (h)	9.50*	70.95	11.25*	50.23
t_1/2_ (h)	43.63	56.47	31.34	43.50
AUC_m_/AUC_p_	–	–	69.79	36.18

AUC_24_; area under the curve from zero to 24 h, AUC; the area under the curve from zero to infinite, C_max_; maximum concentration observed, T_max_; time to reach C_max_ t_1/2_; elimination half-life, V_ss_; volume of distribution at steady state, AUC_m_/AUC_p_; ratio of metabolite to the parent drug. Parameters expressed as an estimate with a percentage of percentage of coefficient of variation. *Significant difference between micellar and oil formulation (*p* < 0.05).

**Figure 5:**
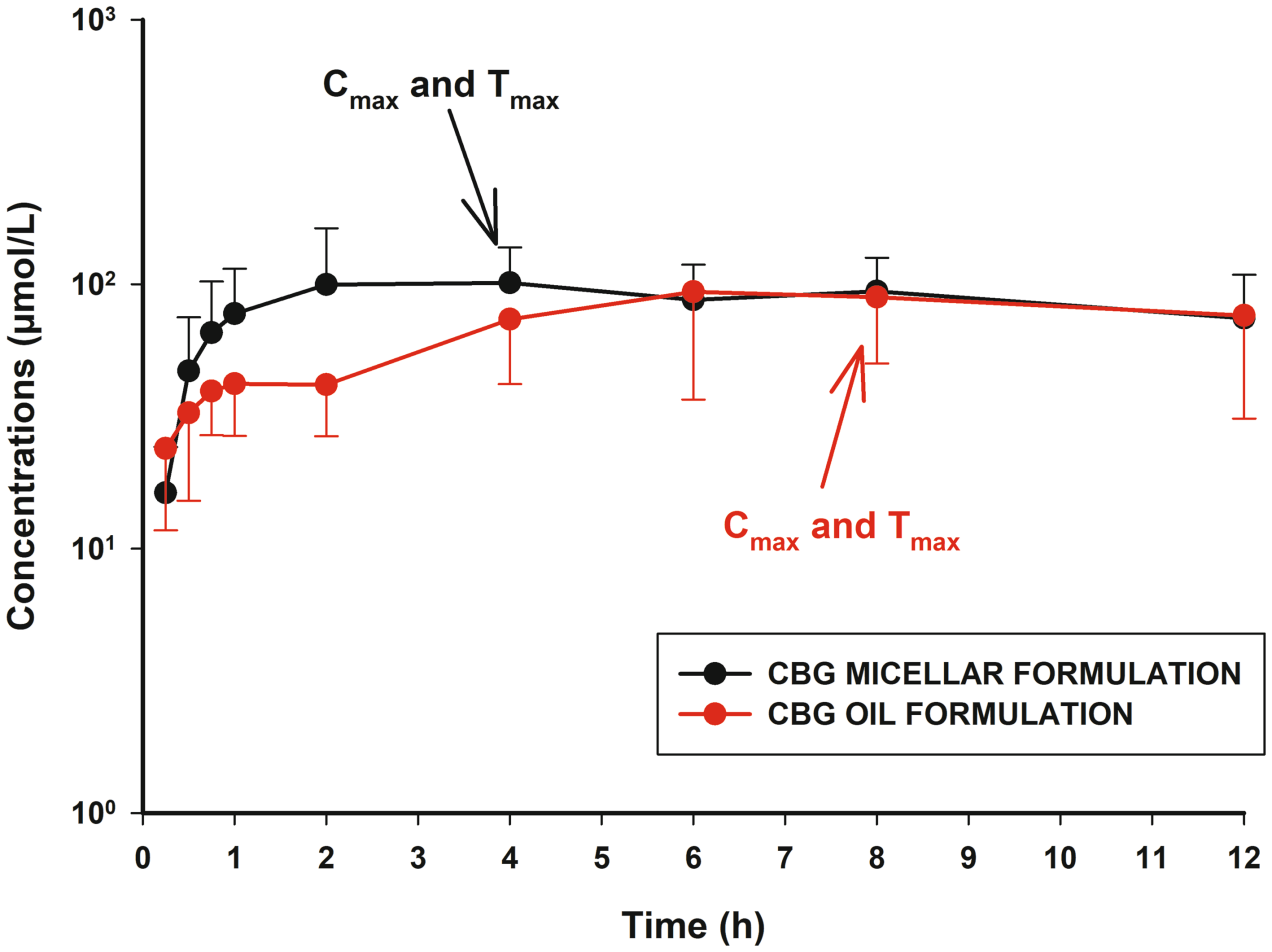
Plot of plasma concentrations (mean ± SD) of cannabigerol (CBG) in horses (*n* = 8) up to 12 h after oral administration in micellar formulation (black line), and oral administration in oil formulation (red line). C_max_ and T_max_ values are plotted for both treatments to facilitate comparative analysis between absorption profiles.

The absorption of CBG after oral administration was adequately described using a Weibull function for both formulations (34). Furthermore, the inclusion of the type of formulation as a categorical covariate improved the estimation of the absorption rate constant (ka) and the shape factor (*p* < 0.05; Table 1). In this way, T_max_ was significantly lower for the micellar formulation, indicating faster absorption compared to the oil formulation, while C_max_ values were similar from a statistical point of view (Figure 5; Table 2; Supplementary Figure S11).

The estimated oral bioavailability was 28% and differences between formulations were not found (Table 1). AUC_24_ and total AUC values were also comparable.

Terminal half-lives for IV and oral routes ranged between 29 and 46 h (Table 2) but were not significantly different from a statistical point of view. However, the highest values were observed with the oil formulation (*p* > 0.05). Regarding the volume of distribution, a high value of V_ss_ of 74 L/kg was obtained.

The formation of the CBG-G metabolite can be observed in Figure 2 and parameterized in Tables 1, 2, respectively. The glucuronide levels were higher than the parent drug concentrations, with AUC_m_/AUC_p_ ratios ranging from 54 to 70. These high concentrations were explained by a lower volume of distribution and a single compartment with 75% of biotransformation to the metabolite (35). Half-lives were close to 21 and 31 h between doses and routes, and no differences were found. Moreover, the CBG-G levels showed shorter T_max_ values for micellar formulation in comparison to oil formulation but with similar AUC_24_, AUC, and C_max_ values. The conversion of the drug described by the AUC_m_/AUC_p_ ratios was higher for the oil formulation but was not statistically different from the micellar formulation or the IV route (p > 0.05).

**Figure 6:**
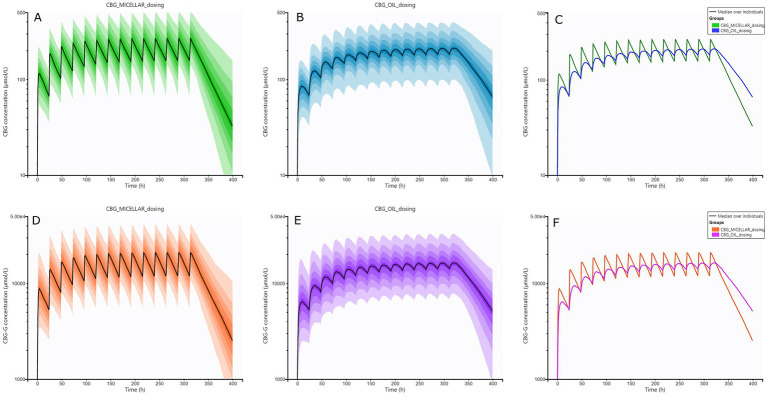
Plots of simulated plasma concentrations of cannabigerol (CBG) and its metabolite CBG-glucuronide (CBG-G) in horses after multiple oral administrations of CBG. Oral treatments using micellar and oil formulations every 24 h for 14 days were simulated. **(A)**: CBG concentrations by micellar formulation (green), **(B)**: CBG concentrations by oil formulation (blue), **(C)**: combined median CBG concentrations by micellar (green) and oil (blue) oral administrations. **(D)**: CBG-G concentrations by micellar formulation (green), **(E)**: CBG-G concentrations by oil formulation (blue), **(F)**: combined median CBG-G concentrations by micellar (green) and oil (blue) oral administrations.

The pharmacokinetic parameters of CBG and CBG-G after oral simulated doses of CBG at 10 mg/kg each 24 h for 14 days for micellar and oil formulations are presented in Table 3 and Figure 6, respectively. It can be observed that the absorption curves did not show the same profiles for the first dose and for the stationary state, which could be reached from the fifth or sixth day, approximately. Statistical comparison of these three parameters indicated differences between formulations in C_max_, T_max_, and AUC values (*p* < 0.05) (Table 3; Supplementary Figures S12–S14).

**Table 3:** Simulated pharmacokinetics parameters of CBG after oral doses at 10 mg/kg each 24 h for 14 days for micellar and oil formulations, resepectively (*n* = 5,000).

Formulation	Parameter	CBG		CBG-G	
		Estimate	% CV	Estimate	% CV
Micellar	AUC_24_ (μmol/L·h)	2281.7	38.89	185128.28	54.29
Micellar	AUC_ss_ (μmol/L·h)	5225.31	40.95	414732.88	46.75
Micellar	Accumulation ratio	2.35	25.59	2.38	25.67
Oil	AUC_24_ (μmol/L·h)	1901.64*	40.86	158703.12*	61.35
Oil	AUC_ss_ (μmol/L·h)	4619.62*	38.63	370173.09*	47.07
Oil	Accumulation ratio	2.54	28.15	2.57	28.38

Formulation	Parameter	CBG		CBG-G	
		Estimate	% CV	Estimate	% CV
Micellar	C_max_ (μmol/L)	125.25	42.68	10227.83	60.72
Micellar	C_max_ss_ (μmol/L)	281.86	38.14	22364.13	47.55
Micellar	T_max_ (h)	2.99	55.68	3.65	45.98
Micellar	T_max_ss_ (h)	121.26	0.76	121.67	0.95
Oil	C_max_ (μmol/L)	93.11*	42.89	7912.51*	65.88
Oil	C_max_ss_ (μmol/L)	206.48*	39.46	16624.39*	49.39
Oil	T_max_ (h)	9.87*	41.52	10.27*	39.58
Oil	T_max_ss_ (h)	130.6*	3.41	131*	3.38

AUC_24_; area under the curve from zero to 24 h, AUC_ss_; the area under the curve at steady state, C_max_; maximum concentration observed from zero to 24 h, T_max_; time to reach C_max_, C_max_ss_; maximum concentration observed at steady state, T_max_ss_; time to reach C_max_ss_. Parameter expressed as an estimate with a percentage of percentage of coefficient of variation. *Significant difference between micellar and oil formulation (*p* < 0.05).

## Discussion

In recent years, the use of cannabinoids has gained popularity in veterinary medicine, with CBD as the main drug investigated (36). However, following recent studies, another related molecule, CBG, could have a promising future in equine patients (13). In this context, and because their PK properties in the horse are unknown, studies describing their disposition after IV and oral administration is mandatory (22).

To our knowledge, this is the first study determining the *in vivo* metabolism and PK of CBG in horses following IV and oral administrations using micellar and oil-based formulations. In general, the results derived from our research suggest firstly, a high biotransformation of CBG to phase I and phase II metabolites, with glucuronide as the main metabolite (CBG-G); secondly, an extensive tissue distribution with high clearance and long half-life after IV and oral administration; thirdly, an oral bioavailability of 28% similar between formulations; fourthly, different absorption profiles with the micellar formulation showing a faster absorption rate; and fifthly, the simulations produced different concentration vs. time curve, with higher C_max_ and shorter T_max_ from the micellar formulation and a prolonged plasma exposure for the oil-based formulation.

Our first objective was to determine the *in vivo* metabolism of CBG and to propose the pathways of biotransformation. In this context, CBG had an extensive hepatic biotransformation, with the formation of epoxide and hydroxide metabolites from phase I, and a glucuronide metabolite from phase II (Figure 1). Considering the LC–MS/MS data, two main phase I pathways were proposed: one involving hydroxylation at position 8′, followed by reduction between carbons 6′-7′; and a second one starting with epoxidation at position 6′-7′, followed by hydroxylation to generate 6′,7′-dihydroxy-CBG, and final reduction at position 2′-3′. In phase II, CBG underwent direct glucuronidation to form the conjugate CBG-G.

*In vitro* studies using microsomes from humans and other species, such as cats or rabbits, have described closely related pathways, suggesting that the biotransformation of CBG includes similar metabolites between species but with different amounts (14, 15, 37, 38). In humans, the main subclasses of cytochrome P450 (CYP) and uridine diphospho-glucuronosyltransferase (UGT) enzymes involved in CBG metabolism are thought to be CYP3A4, CYP2D6, and CYP2C9, and UGT1A1, UGT2A1, UGT2B4, and UGT2B7, respectively (15, 38). However, to our knowledge, the equine orthologs have not been identified for CBG, but subtypes such as CYP2C92, CYP2D50, or UGT2B31 obtained from *in vitro* studies with other drugs in horses could be involved, but further studies in this regard would be necessary to confirm this hypothesis (39–41).

Our second objective was to determine the main PK parameters of CBG in horses. The concentration vs. time curve observed was well described with a two-compartment model for each route and dose tested (Table 1; Figure 3).

During model development, some random effects showed high coefficients of variation and were removed to improve the stability of the model. This approach reduced potential overparameterization without altering the estimation of the final parameters (16). These modifications, together with shrinkage values and goodness-of-fit diagnostics plots, suggest that the PK model adequately captured the interindividual variability for CBG and CBG-G compounds (32, 33).

After IV administration, CBG showed an extensive tissue penetration with a V_ss_ of 74 L/kg; this observation suggests a broad distribution of the drug between plasma and tissues (42, 43). Related values have been observed in other highly lipophilic drugs administered to equines, such as bromhexine, amiodarone or CBD, at 33, 31, and 36 L/kg, respectively (16, 44, 45). This high distribution may be associated with a long elimination half-life, which could also be a relevant factor for CBG and its potential accumulation into the tissues in prolonged treatments, as has been observed in recent assays in equines with CBD at multiple doses (46, 47).

The Cl estimated for CBG was 1.67 L/h/kg. Based on a cardiac output of 3.38 L/h/kg, this corresponds to a high extraction ratio of 0.49. According to previously established veterinary breakpoints, CBG can be classified as a drug with high clearance in horses. This value is also comparable to that reported for CBD in equines (1.46 L/h/kg) (48). This result suggests that the elimination of this cannabinoid was mediated by an intensive hepatic metabolism, and following the observed patterns in other mammalian species, glucuronidation represents the main metabolic route with a percentage of biotransformation ranging from 73 to 76% (14, 35). These observations are consistent with the AUC_m_/AUC_p_ ratios obtained (Table 2) and indicate that this phase II reaction is highly efficient in horses, contributing to the rapid elimination of the parent compound (35). In human medicine, levels of CBG-G have been suggested as a biomarker after inhalation of cannabis, indicating its relevance as a metabolite (49, 50). These findings highlight the importance of the detection of metabolites and their inclusion in NLME models to use in equine practice, as has been recommended (51, 52).

The half-life of CBG was approximately 29.22 h, whereas that for CBG-G was 21.01 h after IV administration. This trend was maintained after oral administration, where the half-life ranged between 28.58 and 43.63 h, and for CBG-G was 23.56 and 31.34 h for micellar and oil formulations, respectively. These results indicate that in comparison to CBD, CBG exhibits a longer half-life in horses (16). These data are consistent with the high V_ss_ achieved in this study, suggesting that, despite the high Cl observed, this drug is widely distributed in tissues before elimination, and that could also be influenced by its high lipophilicity, promoting its redistribution (53). Compared with other species, this half-life is considerably longer than that reported after oral or intraperitoneal administration in dogs (1–2 h), rats (9.3 h), mice (2.6 h), or humans (3–5 h) (10–12). However, it is important to note that half-life is a secondary parameter highly influenced by the drug’s disposition, formulations administered, analytical methods used for the determination of concentrations, and the species investigated. Therefore, comparisons of this parameter should be cautious, taking into account all sources of variability (53, 54).

The third objective of this research was to evaluate the oral absorption of CBG and the bioavailability of the two formulations tested. In this context, the oral bioavailability of CBG was estimated at 28%, with no significant differences between the micellar and oil-based formulations. This percentage is higher than that reported for CBD in horses (7.9–14%), depending on the formulation and dose used (16, 55), suggesting that CBG might be more efficiently absorbed in the equine gastrointestinal tract (18, 20).

As a Class II compound in the Biopharmaceutical Classification System (BCS), CBG has low aqueous solubility and high permeability. It is also classified as Class II in the Biopharmaceutics Drug Disposition Classification System (BDDCS), indicating extensive first-pass metabolism (56). These classifications might suggest that the absorption of CBG can be enhanced by lipid-based formulations, such as oils or micelles, which improve the solubility into the intestinal lumen in the same way as CBD (16, 57).

Nonetheless, and beyond these properties, the interaction with transporters may influence the bioavailability. Recent studies have reported that cannabinoids interact with the breast cancer resistance proteins (BCRP) located in the enterocytes, as well as with p-glycoprotein (P-gp), which could limit their systemic absorption (58). The effect of these proteins on the absorption of cannabinoids is still poorly understood, and further research is needed to evaluate the action of transporters in the equine context (18, 59). Nevertheless, our results are correlated with clinical observations. In a recent study, an oral CBG/CBD and cannabidiolic acid (CBDA) mixture was administered to horses with chronic (OA), resulting in improvements in pain indicators without adverse effects. Although drug concentrations were not measured, these findings suggest an effective absorption of CBG in equines, aligning with the bioavailability observed in our study compared to CBD (13, 16).

After evaluation of the absorption between oral formulations, no differences were found in the bioavailability and the C_max_ values (Table 2). However, the micellar formulation displayed a lower T_max_ and higher k_a_ (Figure 4). These findings are consistent with previous studies in which micellar formulations enhanced the absorption rate without necessarily increasing total bioavailability (16). The Weibull absorption model applied in this study showed that the micellar formulation had a higher *β* parameter of 1.59 (Table 1; Figure 5), indicating a more pronounced absorption phase due to improved drug release compared to lipids such as the oil formulation (60, 61). In this context, the use of Weibull functions is particularly useful to explain complex absorption processes that do not follow first-order kinetics and is especially relevant for formulations with absorption changes over time due to factors such as progressive drug dissolution or delayed release mechanisms (34). Consequently, the type of formulation affected the absorption profiles and was retained as a categorical covariate. Similar findings were described with the same formulations and doses in horses using CBD, and although CBG is more lipophilic than CBD, the absorption vehicle can exert an important influence (16). This is true for the oil formulations because the lipids may reduce gastric emptying and increase retention time with long T_max_ values but have no effect on bioavailability (62). In conclusion, while both formulations resulted in comparable systemic exposure, micellar formulations provided faster and more consistent absorption, which may be beneficial for clinical applications requiring rapid onset of action, while the oil formulation could be more suitable to achieve more sustained levels over time, warranting further research into its clinical applications in equines.

The fourth objective of this research was to simulate different CBG concentrations with the two formulations tested and compare the oral profiles after multiple oral CBG doses every 24 h for 2 weeks. After simulating and comparing the parameters, differences in absorption and disposition were observed. Firstly, the micellar formulation exhibited significantly faster absorption compared to the oil formulation, as evidenced by lower T_max_ values in the micellar group at the first dose and stationary state, showing that micelles could facilitate earlier CBG absorption (Figure 5; Table 3). Secondly, the simulated C_max_ values at first dose and into the stationary state were higher than the oil formulation for both CBG and CBG-G, and thirdly, the AUC at the first dose and the stationary state also were larger and different from a statistical point of view for the micellar formulation. The result suggests that the increased absorption rate may also translate into higher plasma concentrations in some individuals, as has been indicated (16). Moreover, it is important to consider that the differences detected in these simulations were more pronounced than in the individual data described due to the larger number of simulated subjects in comparison to the observed data (24, 33, 63). These considerations may explain why the simulations could detect significant differences in C_max_ and T_max,_ whereas in the experimental study, statistical significance was only observed for T_max_ (22, 64).

From a clinical perspective, these differences between formulations could be relevant in therapeutic scenarios requiring a faster action for micellar or a more sustained action for the oil formulation, as has been described for CBD (16, 65). Additionally, previous studies with related lipophilic drugs have reported that micellar formulations improve absorption and reduce interindividual variability, which aligns with our findings and could explain the observations of the simulations (66). Finally, the predicted concentrations in our research should be taken with caution because they were derived from a reduced population from a preclinical assay (8 animals per trial), but larger animal populations in a clinical context should be studied to evaluate their usefulness in clinical studies (16, 22). Nevertheless, the results derived from this study offer important insights into the use of CBG in horses and could represent a foundation for future trials involving different clinical scenarios (51).

In conclusion, this study describes the *in vivo* metabolism and pharmacokinetics of CBG and the main metabolite glucuronide in horses, emphasizing the impact of micellar and oil formulations on absorption and systemic exposure, including different scenarios with the Monte Carlo simulation, and supports the development of further studies to explore the use of CBG in equine medicine.

## Data Availability

The raw data supporting the conclusions of this article will be made available by the authors, without undue reservation.
