# Supplementary material for: Metabolism, pharmacokinetics, and bioavailability of cannabigerol in horses following intravenous and oral administration with micellar and oil formulations
Source: Front Vet Sci. 2025 Oct 29;12:1688214. doi: 10.3389/fvets.2025.1688214 (PMC12607281; doi:10.3389/fvets.2025.1688214)
Supplement: Supplementary file 1 [file Data_Sheet_1.docx]

Supplementary Material

# Supplementary Figures and Tables

**CONTENTS**

**Figure S1: Proposed pathway of the biosynthesis of CBG metabolites in horses.**

**Figure S2: chromatogram and MS/MS spectrum of CBG.**

**Figure S3: chromatogram and MS/MS spectrum of CBG-G.**

**Figure S4: chromatogram of main phase I metabolites of CBG.**

**Figure S5: chromatogram of hydroxy and isomers of phase I metabolites of CBG.**

**Figure S6: chromatogram of tentative minority phase I metabolites of CBG.**

**Code of the PK model in MLXTRAN language.**

**Table S1. Extended PK by NLME model.**

**Table S2. Extended PK of secondary parameters.**

**Table S3. Extended table for simulated parameters.**

**Table S4. Clinical laboratory values.**

**Figure S7. Observations vs predictions plots for CBG.**

**Figure S8. Scatter plot of the residuals for CBG.**

**Figure S9. Observations vs predictions plots for CBG.**

**Figure S10 Scatter plot of the residuals for CBG.**

**Figure S11. Box plots of categorical covariates by micellar formulation versus oil formulation.**

**Figure S12: Box plots for statistical comparisons between C_max_, T_max_, AUC_24_ and AUC_ss_ of CBG after simulated oral CBG administration for micellar (red) and oil (blue) formulations.**

**Figure S13: Box plots for statistical comparisons between AUC_24_ and AUC_ss_ for CBG-G after simulated oral CBG administration for micellar (red) and oil (blue) formulations.**

**Figure S14: Box plots for statistical comparisons between C_max_ and C_max-ss_, and T_max_ and T_max-ss_ for CBG after simulated oral CBG administration for micellar (red) and oil (blue) formulations.**

**Figure S15: Box plots for statistical comparisons between C_max_ and C_max-ss_, and T_max_ and T_max-ss_ for CBG-G after simulated oral CBG administration for micellar (red) and oil (blue) formulations.**


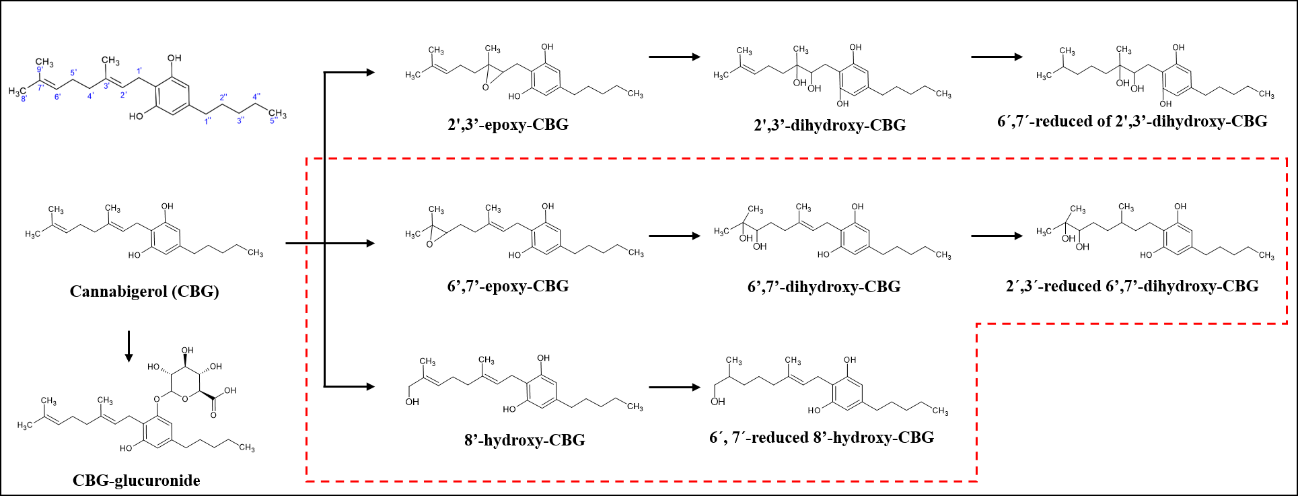


**Supplementary** **Figure 1.** Proposed pathway of the biosynthesis of CBG metabolites in horses. The metabolites enclosed in the dotted line correspond to the most abundant derivatives. The reduced forms were tentatively identified because they exhibit a similar fragmentation pattern to the unsaturated forms, but with an additional mass-to-charge ratio (m/z) of 2.0156 units, corresponding to the addition of two hydrogen atoms.


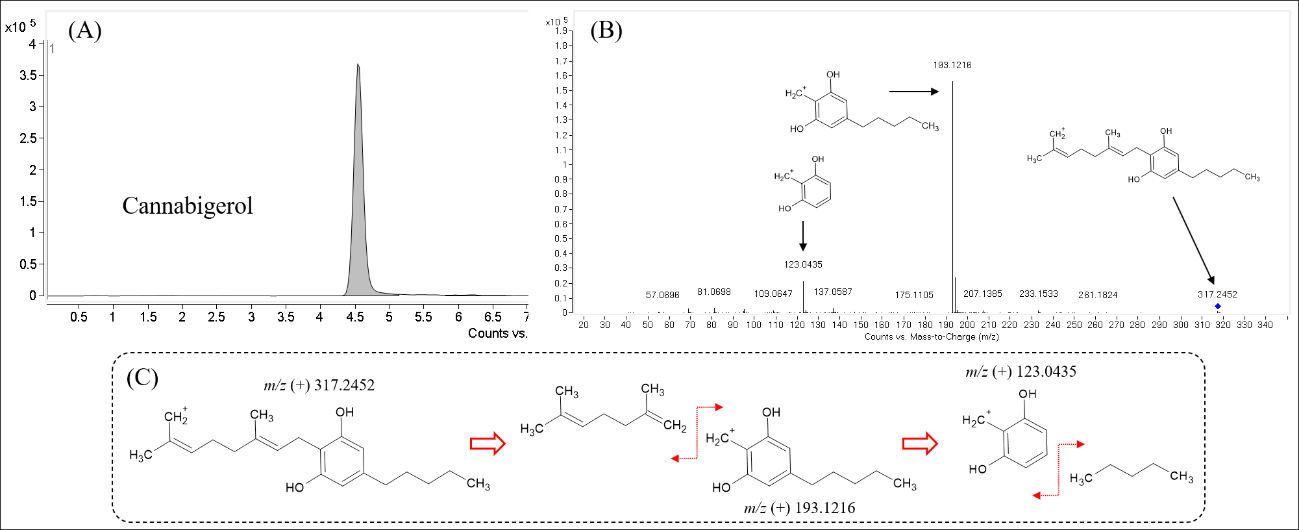


**Supplementary Figure 2.** Extracted ion chromatogram (A), MS/MS spectrum (B) and fragmentation scheme of cannabigerol (CBG) in positive ion mode.


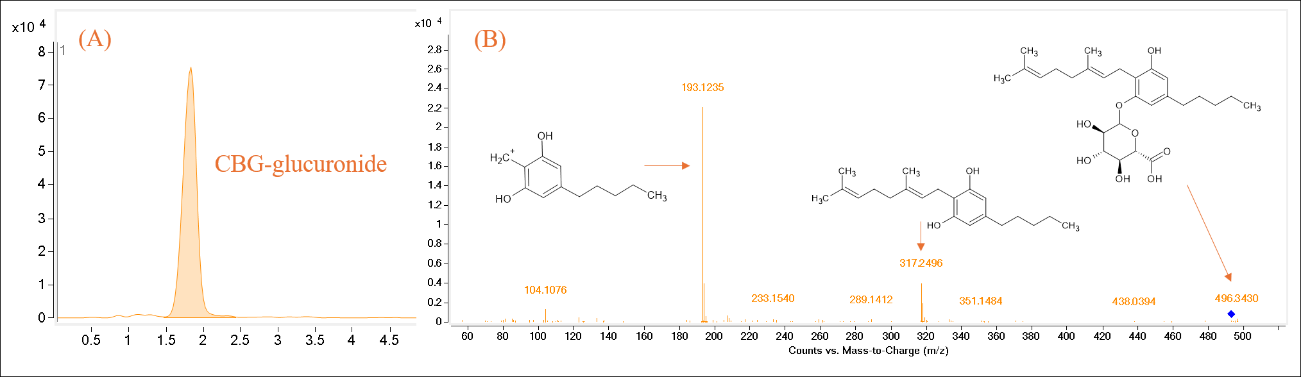


**Supplementary Figure 3**. Extracted ion chromatogram (A) and MS/MS spectrum (B) of cannabigerol glucuronide in positive ion mode.


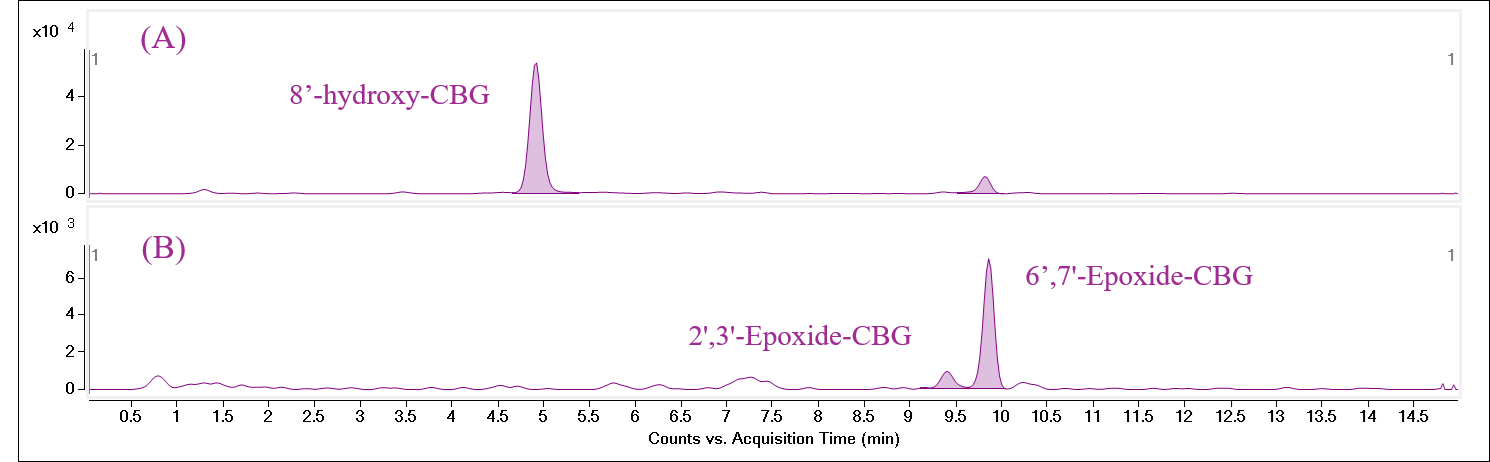


**Supplementary Figure 4.** Extracted ion chromatogram of 8’-hydroxy-CBG (A), and 2',3'-Epoxide-CBG and 6',7'-Epoxide-CBG isomers (B).


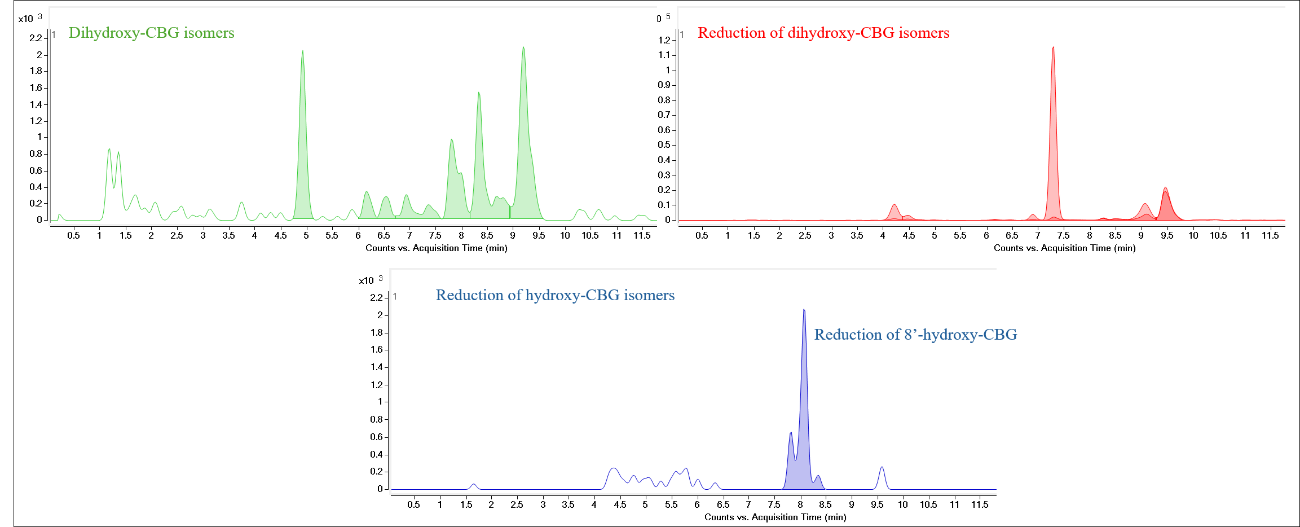


**Supplementary Figure 5.** Extracted ion chromatograms of dihydroxy-CBG isomers and the reduced forms of both dihydroxy-CBG and hydroxy-CBG isomers.


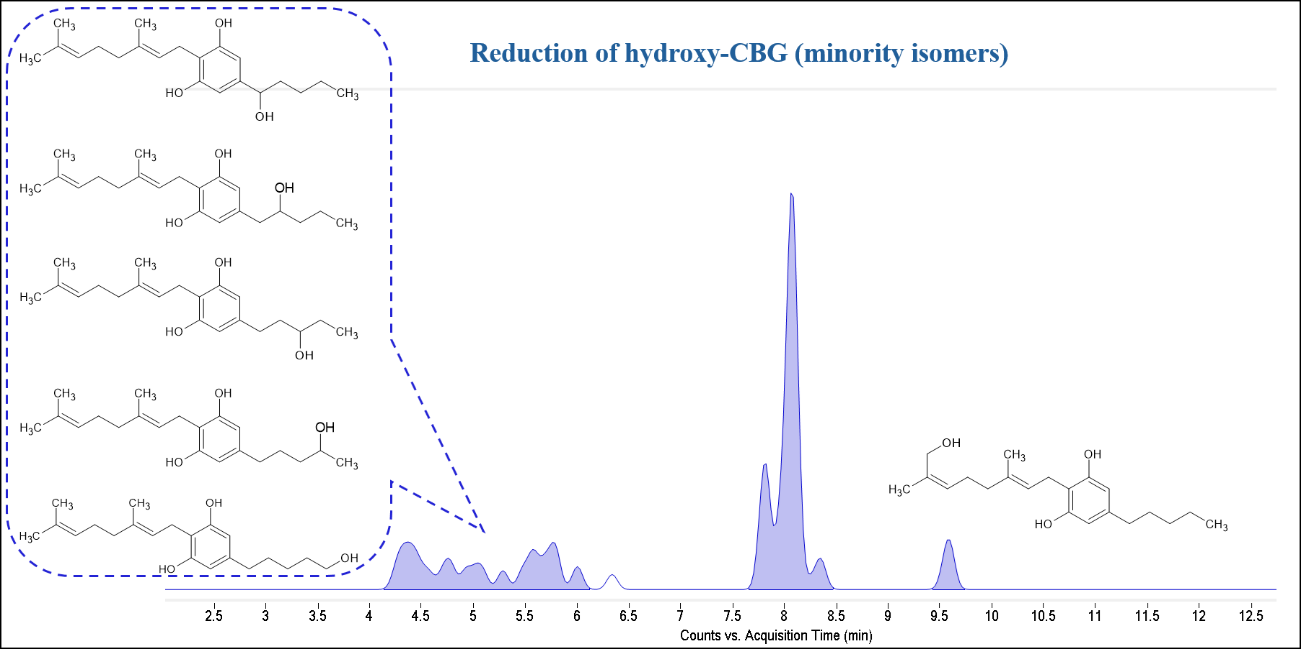


**Supplementary Figure 6. T**entatively extracted ion chromatograms of reduced forms of hydroxy-CBG minority isomers.

DESCRIPTION: PK MODEL FOR CBG AND IS MAIN METABOLITE CBG-G AFTER INTRAVENOUS AND ORAL ADMINISTRATION OF CBG

[LONGITUDINAL]

input = {F, ka, beta, Cl, V1, Q2, V2, Vm, Fm, Clm}

PK:

depot(adm=1, target=A1) ; IV administration for CBG

depot(adm=2, target=A4, p=F) ; oral administration for CBG

EQUATION:

odeType = stiff

; Initial conditions

t_0=0

A1_0=0 ; IV central compartment for CBG

A2_0=0 ; Peripheral compartment for CBG

A3_0=0 ; Glucuronide metabolite CBG-G

A4_0=0 ; oral administration of CBG(micellar, oil)

; Parameter transformations, definition of Weibull absorption

kaw = ka*beta*((max(t,0)*ka)^(beta-1))

; ODE system

ddt_A1 = kaw*A4 - Cl*A1/V1 - Q2/V1*A1 + Q2/V2*A2

ddt_A2 = Q2/V1*A1 - Q2/V2*A2

ddt_A3 = Cl*Fm*A1/V1 - Clm*A3/Vm

ddt_A4 = - kaw*A4

; Concentrations

C1 = A1/V1 ; Plasma concentrations at central compartment

C2 = A2/V2 ; Peripheral concentrations 1

C3 = A3/Vm ; Glucuronide concentrations

; Area under the curve

AUC_0 = 0

ddt_AUC = C1

AUC24_0 = 0

if(t < 24)

dAUC24 = 1/V1 * A1

else

dAUC24 = 0

end

ddt_AUC24 = dAUC24

AUCC3_0 = 0

ddt_AUCC3 = C3

AUC24C3_0 = 0

if(t < 24)

dAUC24C3 = 1/Vm * A3

else

dAUC24C3 = 0

end

ddt_AUC24C3 = dAUC24C3

OUTPUT:

output = {C1, C3}

table = {AUC, AUCC3, AUC24, AUC24C3}

**TABLE 1:**

Extended table for pharmacokinetic parameters of CBG and CBG-G in horses after intravenous CBG at 1.0 mg/kg and oral CBG at 10.0 mg/kg by micellar and oil formulations, respectively.

|  | NLME estimates | | | Boostrap analysis | | | Shrinkage |
| --- | --- | --- | --- | --- | --- | --- | --- |
| Parameter | Value | P2.5 | P97.5 | Median | P2.5 | P97.5 | (%) |
| F | 0.28 | 0.22 | 0.34 | 0.29 | 0.25 | 0.32 | -3.54 |
| ka (1/h) | 0.99 | 0.65 | 1.53 | 0.95 | 0.54 | 1.1 | 1.08 |
| Cov_ka_formulation_ | -1.18 | -1.65 | -0.71 | -1.22 | -1.68 | -0.61 |  |
| β | 1.59 | 1.24 | 2.03 | 1.43 | 1.16 | 1.94 | 1.4 |
| Cov_γ_formulation_ | -0.82 | -1.06 | -0.58 | -0.77 | -1.14 | -0.57 |  |
| Cl (l/h/kg) | 1.67 | 1.36 | 2.05 | 1.75 | 1.37 | 2.04 | -3.03 |
| V_c_ (L/kg) | 32.15 | 17.54 | 58.93 | 43 | 10.87 | 69.78 | -4.85 |
| Q (L/h/kg) | 154.5 | 73 | 326.99 | 82.35 | 3.98 | 243.5 | - |
| V_p_ (L/kg) | 36.12 | 23.96 | 54.43 | 41.29 | 16.35 | 58.26 | 6.25 |
| V_m_ (L/kg) | 0.0047 | 0.003 | 0.0075 | 0.0041 | 0.0015 | 0.0097 | -14.4 |
| F_m_ | 0.75 | - | - |  |  |  | - |
| Cl_m_ (L/h/kg) | 0.016 | 0.013 | 0.021 | 0.016 | 0.014 | 0.023 | -5.85 |
| Fixed Effects by Category | | | | | | | |
| ka__micellar formulation_ (1/h) | 0.99 | 0.65 | 1.53 |  |  |  |  |
| β__micellar formulation_ | 1.59 | 1.24 | 2.03 |  |  |  |  |
| ka__oil formulation_ (1/h) | 0.31 | 0.19 | 0.5 |  |  |  |  |
| β__oil formulation_ | 0.7 | 0.54 | 0.91 |  |  |  |  |
|  |  |  |  |  |  |  |  |
| Random Effects CV(%) | | | |  | Residural error | | |
| IIV_F | 25.52 | IOV_F | 13.93 |  | b1 | 0.29 |  |
| IIV_ka | 46.64 | IOV_ka | 37.99 |  | b2 | 0.41 |  |
| IIV_β | 26.01 | IOV_β | 12.8 |  |  |  |  |
| IIV_Cl | 27.95 |  |  |  |  |  |  |
| IIV_V_C_ | 66.74 |  |  |  |  |  |  |
| IIV_V_P_ | 21.22 |  |  |  |  |  |  |
| IIV_V_m_ | 53.58 |  |  |  |  |  |  |
| IIV_Cl_m_ | 32.23 |  |  |  |  |  |  |

**TABLE 2**:

Extended table for the secondary pharmacokinetic parameters for CBG and CBG-G after intravenous CBG at 1.0 mg/kc and oral administration of 10.00 mg/kg of CBG by oil or micellar formulations, respectively.

| IV administration of CBG at 1.0 mg/kg | | | | | | | | |
| --- | --- | --- | --- | --- | --- | --- | --- | --- |
|  | CBG |  |  |  | CBG-G |  |  |  |
| Parameter | Value | P2.5 | P97.5 | CV(%) | Value | P2.5 | P97.5 | CV(%) |
| AUC_24_ (µmol/L·h) | 855.75 | 428.11 | 1298.36 | 41.45 | 62419.46 | 41094.77 | 89431.21 | 25.49 |
| AUC (µmol/L·h) | 2376.23 | 980.09 | 3651.10 | 46.82 | 105071.97 | 57666.05 | 184625.94 | 42.44 |
| C_max_ (µmol/L) | 74.92 | 42.33 | 107.24 | 31.04 | 5508.28 | 3184.34 | 7721.73 | 31.05 |
| T_max_ (h) | 1.80 | 0.08 | 5.65 | 136.04 | 2.28 | 0.54 | 5.65 | 81.47 |
| t_1/2x_ (h) | 29.22 | 14.27 | 63.66 | 62.89 | 21.01 | 7.57 | 48.01 | 69.23 |
| V_ss_ (L/kg) | 74.31 | 44.84 | 100.60 | 27.69 | - | - | - | - |
| AUC_m_/AUC_p_ | - | - | - | - | 54.25 | 18.15 | 107.70 | 58.26 |
| Oral administration of CBG at 10.0 mg/kg by micellar formulation | | | | | | | | |
|  | CBG | | | | CBG-G | | | |
| Parameter | Value | P2.5 | P97.5 | CV(%) | Value | P2.5 | P97.5 | CV(%) |
| AUC_24_ (µmol/L·h) | 1817.02 | 1129.50 | 2411.58 | 27.15 | 138645.04 | 90704.99 | 222624.35 | 33.21 |
| AUC (µmol/L·h) | 4109.79 | 2796.72 | 6268.55 | 31.02 | 247414.22 | 166318.20 | 358258.57 | 26.80 |
| C_max_ (µmol/L) | 132.70 | 85.11 | 220.72 | 36.66 | 10400.83 | 7255.67 | 15556.97 | 29.24 |
| T_max_ (h) | 4.00 | 2.00 | 6.00 | 46.29 | 5.75 | 4.00 | 8.00 | 29.03 |
| t_1/2x_ (h) | 28.58 | 18.09 | 38.36 | 30.39 | 23.56 | 12.27 | 54.38 | 66.53 |
| AUC_m_/AUC_p_ | - | - | - | - | 64.41 | 37.04 | 94.05 | 35.56 |
| Oral administration of CBG at 10.0 mg/kg by oil formulation | | | | | | | | |
|  | CBG | | | | CBG-G | | | |
|  | Value | P2.5 | P97.5 | CV(%) | Value | P2.5 | P97.5 | CV(%) |
| AUC_24_ (µmol/L·h) | 1635.11 | 486.63 | 2598.82 | 48.24 | 133925.40 | 74319.05 | 180650.92 | 31.00 |
| AUC (µmol/L·h) | 5136.01 | 2636.62 | 8904.38 | 43.87 | 306448.70 | 153370.91 | 410339.26 | 33.14 |
| C_max_ (µmol/L) | 101.38 | 33.65 | 156.87 | 46.55 | 8003.28 | 4243.13 | 10908.79 | 33.24 |
| T_max_ (h) | 9.50 | 2.70 | 21.90 | 70.95 | 11.25 | 6.35 | 21.90 | 50.23 |
| t_1/2x_ (h) | 43.63 | 19.49 | 90.03 | 56.47 | 31.34 | 20.26 | 51.67 | 43.50 |
| AUC_m_/AUC_p_ | - | - | - |  | 69.79 | 41.17 | 106.94 | 36.18 |

**TABLE 3**

Extended table for the simulated pharmacokinetics parameters of CBG after oral doses at 10 mg/kg each 24 h for 14 days for micellar and oil formulations, receptively (n = 5000).

|  | CBG-G | | | | CBG-G | | | |
| --- | --- | --- | --- | --- | --- | --- | --- | --- |
| Parameter | Value | P2.5 | P97.5 | %CV | Value | P2.5 | P97.5 | %CV |
| AUC_24micellar formulation_ | 2281.7 | 952.03 | 4300.66 | 38.89 | 185128.28 | 64604.92 | 400884.74 | 54.29 |
| AUC_ss_micellar formulation_ | 5225.31 | 2156.21 | 10294.88 | 40.95 | 414732.88 | 158457.12 | 817194.4 | 46.75 |
| Accumulation ratio | 2.35 | 1.45 | 3.68 | 25.59 | 2.38 | 1.49 | 3.74 | 25.67 |
| AUC_24_oil formulation_ | 1901.64 | 752.99 | 3700.92 | 40.86 | 158703.12 | 47056.94 | 385820 | 61.35 |
| AUC_ss_oil formulation_ | 4619.62 | 1822.6 | 8786.08 | 38.63 | 370173.09 | 146460.52 | 759635.86 | 47.07 |
| Accumulation ratio | 2.54 | 1.47 | 4.17 | 28.15 | 2.57 | 1.48 | 4.26 | 28.38 |
|  |  |  |  |  |  |  |  |  |
|  | CBG | | | | CBG-G | | | |
| Parameter | Value | P2.5 | P97.5 | %CV | Value | P2.5 | P97.5 | %CV |
| C_max_micellar formulation_ | 125.25 | 48.57 | 237.16 | 42.68 | 10227.83 | 3269.79 | 24504.18 | 60.72 |
| C_max_ss_micellar formulation_ | 281.86 | 120.24 | 530.54 | 38.14 | 22364.13 | 8251.22 | 44102.16 | 47.55 |
| T_max_micellar formulation_ | 2.99 | 1 | 7 | 55.68 | 3.65 | 1 | 8 | 45.98 |
| T_max_ss_micellar formulation_ | 121.26 | 121 | 124 | 0.76 | 121.67 | 121 | 124.53 | 0.95 |
| C_max_oil formulation_ | 93.11 | 36.23 | 187.37 | 42.89 | 7912.51 | 2290.96 | 20143.93 | 65.88 |
| C_max_ss_oil formulation_ | 206.48 | 81.07 | 400.41 | 39.46 | 16624.39 | 6304.71 | 35340.27 | 49.39 |
| T_max_oil formulation_ | 9.87 | 4 | 20 | 41.52 | 10.27 | 5 | 20 | 39.58 |
| T_max_ss_oil formulation_ | 130.6 | 123 | 143 | 3.41 | 131 | 123 | 143 | 3.38 |

**TABLE 4**

Clinical laboratory values before and 72 hours after administration of cannabigerol (CBG) in horses. Data presented as mean with range.

|  | Before administration | 72 h post administration |
| --- | --- | --- |
|  | IV CBG at 1.00 mg/kg | IV CBG at 1.00 mg/kg |
| Urea (mg/dL) | 26.7 (24 – 32) | 32.30 (40 - 32) |
| Creatinine (mg/dL) | 1.03 (0.87 - 1.53) | 1.00 (0.80 - 1.28) |
| GLDH (IU/L) | 2.96 (2.50 - 5.30) | 2.80 (2.10 - 4.10) |
| AST (IU/L) | 214.3 (204 - 305) | 318.50 (270 - 366) |
| RBC (x10^6^/µL) | 7.22 (6.19 - 7.58) | 6.91 (6.48 - 9.00) |
| Ht (%) | 33.88 (29.30 - 36.8) | 37.54 (34.10 - 39.4) |
| Hb (g/dL) | 11.97 (10.10 - 14.9) | 10.52 (11.26 - 13.5) |
| WBC (x10^3^/µL) | 7.72 (6.82 - 8.2) | 6.98 (6.33 - 7.84) |
| PLT (x10^3^/µL) | 190.22 (93 - 172) | 105.57 (102 - 204) |
|  | | |
|  | Before administration | 72 h post administration |
|  | Oral micellar CBG at 10.00 mg/kg | Oral micellar CBG at 10.00 mg/kg |
| Urea (mg/dL) | 35.50 (26 - 51) | 32.00 (19 - 45) |
| Creatinine (mg/dL) | 1.00 (0.77 - 1.11) | 0.92 (0.70 - 1.0) |
| GLDH (IU/L) | 3.09 (1.90 - 6.90) | 2.57 (1.80 - 3.10) |
| AST (IU/L) | 302.10 (286 - 401) | 300.80 (297 - 451) |
| RBC (x10^6^/µL) | 7.87 (5.97 - 7.99) | 6.64 (5.99 - 9.68) |
| Ht (%) | 35.65 (30.90 - 38.0) | 37.28 (33.50 - 45.2) |
| Hb (g/dL) | 12.80 (10.90 - 14.3) | 14.56 (10.70 - 17.2) |
| WBC (x10^3^/µL) | 8.43 (6.77 - 9.02) | 7.06 (6.28 - 9.24) |
| PLT (x10^3^/µL) | 134.27 (124 - 185) | 200.29 (172 - 260) |
|  | | |
|  | Before administration | 72 h post administration |
|  | Oral OIL CBG at 10.00 mg/kg | Oral OIL CBG at 10.00 mg/kg |
| Urea (mg/dL) | 29.80 (24 - 51) | 34.00 (22 - 47) |
| Creatinine (mg/dL) | 0.95 (0.60 - 1.05) | 0.87 (0.50 -1.00) |
| GLDH (IU/L) | 280 (1.80 - 3.80) | 2.07 (1.60 - 3.10) |
| AST (IU/L) | 290.30 (212 - 430) | 277.10 (261 - 407) |
| RBC (x10^6^/µL) | 7.17 (6.21 - 9.05) | 7.98 (6.17 - 8.91) |
| Ht (%) | 33.48 (31.20 - 42.4) | 37.24 (30.12 - 40.7) |
| Hb (g/dL) | 12.31 (10.00 - 14.9) | 12.12 (10.50 - 14.5) |
| WBC (x10^3^/µL) | 8.09 (7.20 -10.33) | 7.68 (7.01 - 9.10) |
| PLT (x10^3^/µL) | 188.0 (158 - 231) | 164.20 (125 - 287) |

GLDH, glutamate dehydrogenase; AST, aspartate aminotransferase; RBC, red blood cell; Ht, haematocrit; Hb, haemoglobin concentration; WBC, white blood cell; PLT, platelet.


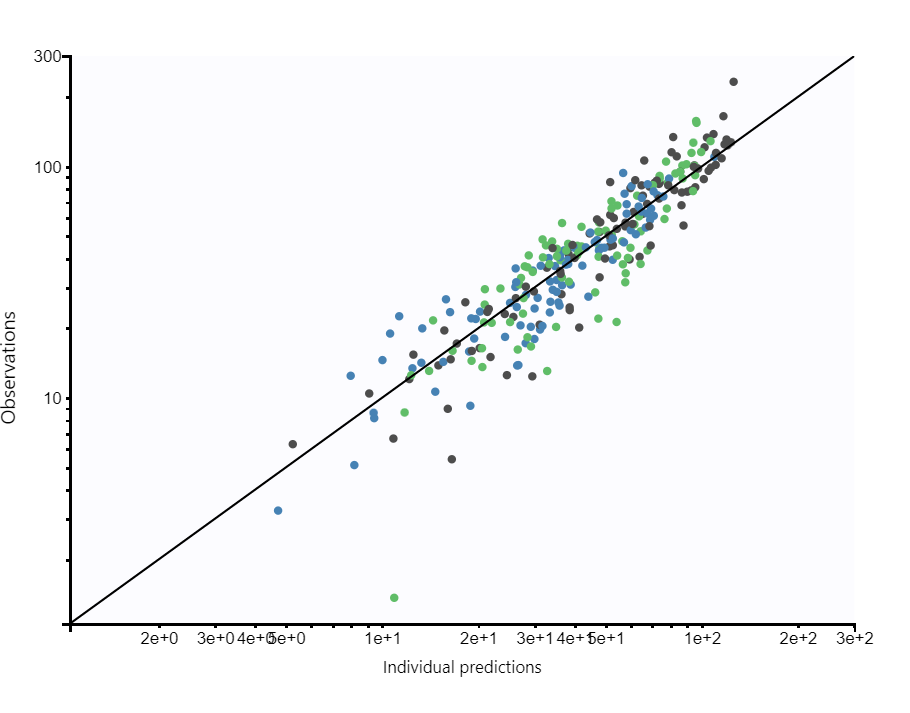


**Supplementary Figure 7.** Observations vs predictions plots for CBG concentrations. Observed concentrations for IV administration are displayed in blue colour, for oral micellar administration in black colour and in green for oral oil administration, respectively.


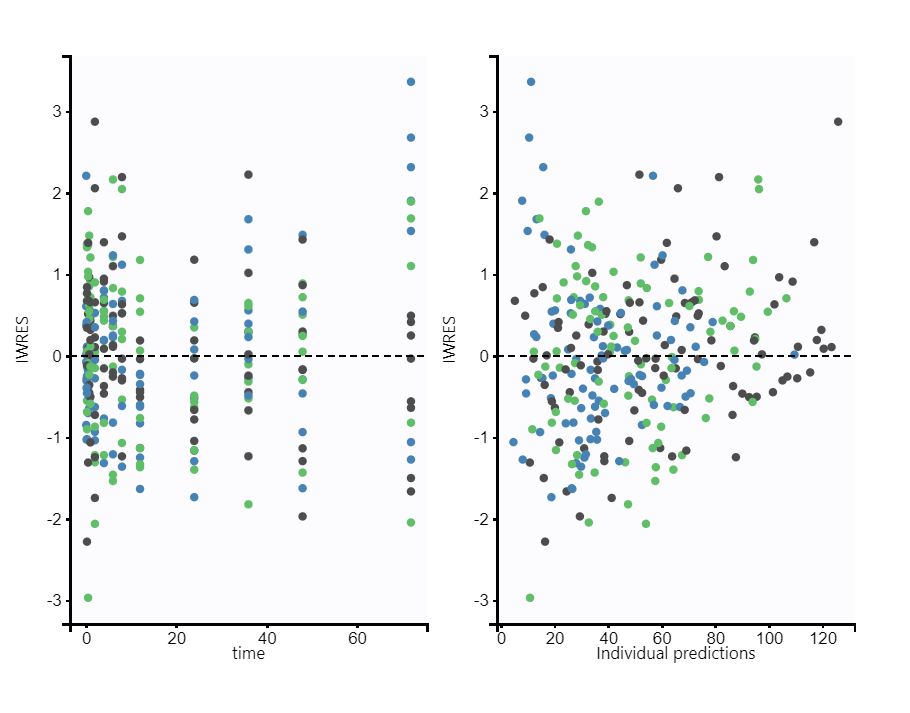


**Supplementary Figure 8.** Scatter plot of the residuals for CBG. Observed data for IV administration are displayed in blue colour, for oral micellar administration in black colour and in green for oral oil administration, respectively.


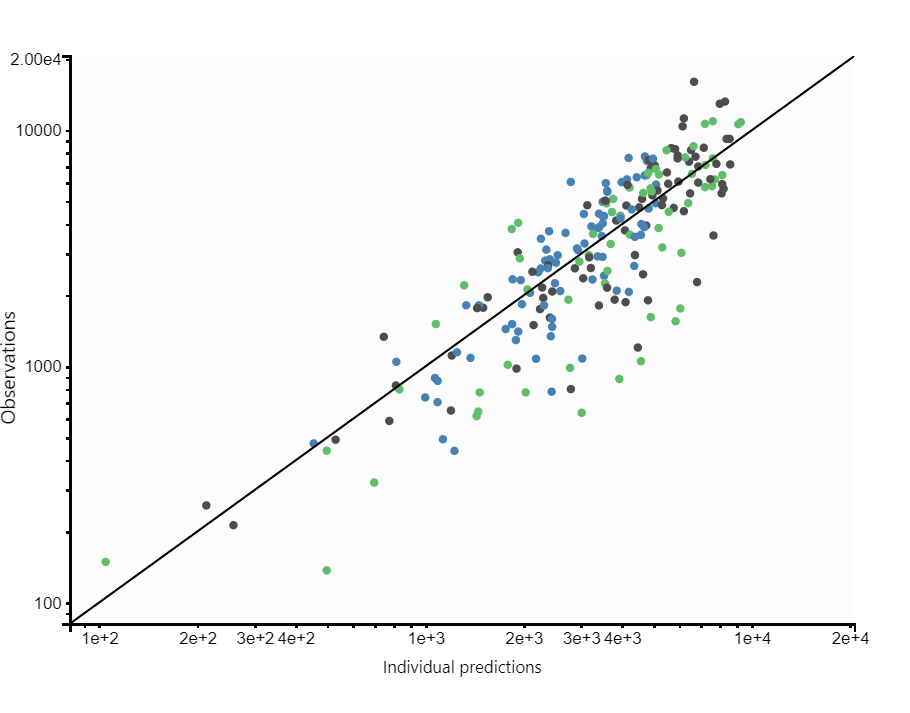


**Supplementary Figure 9.** Observations vs predictions plots for CBG-G concentrations. Observed concentrations for IV administration are displayed in blue colour, for oral micellar administration in black colour and in green for oral oil administration, respectively.


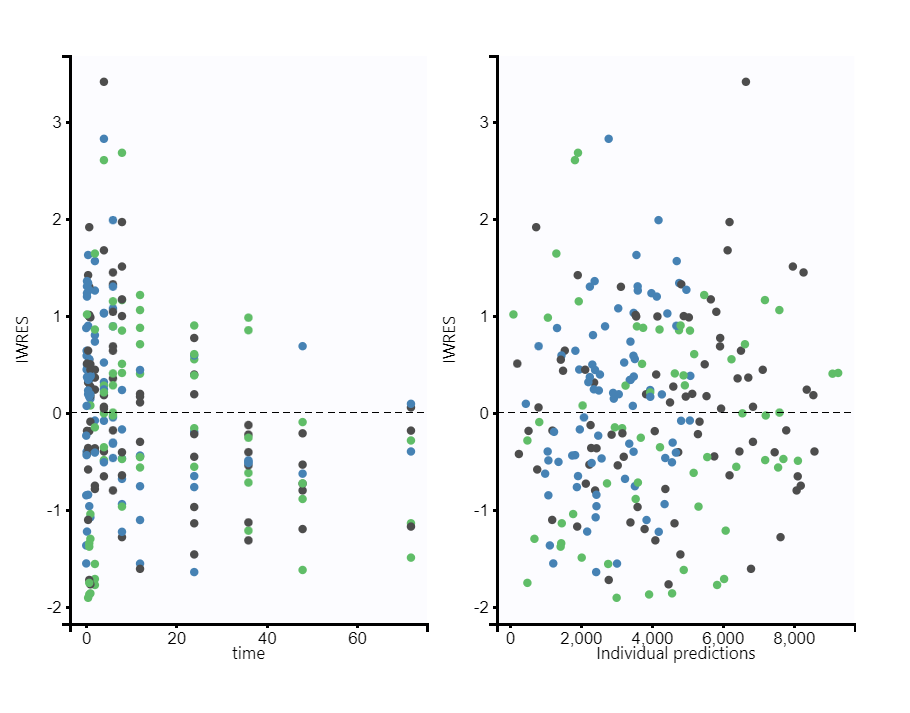


**Supplementary Figure 10.** Scatter plot of the residuals for CBG-G. Observed data for IV administration are displayed in blue colour, for oral micellar administration in black colour and in green for oral oil administration, respectively.


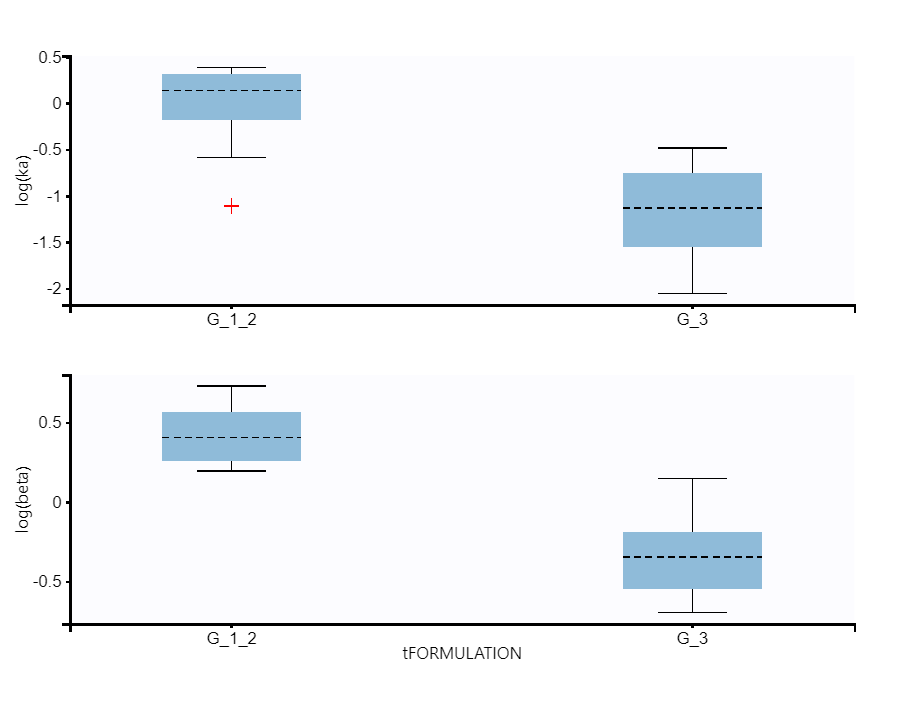


**Supplementary Figure 11.** Box plots of categorical covariates with effect of ka and β in horses: CBG by micellar formulation versus CBG by oil formulation.


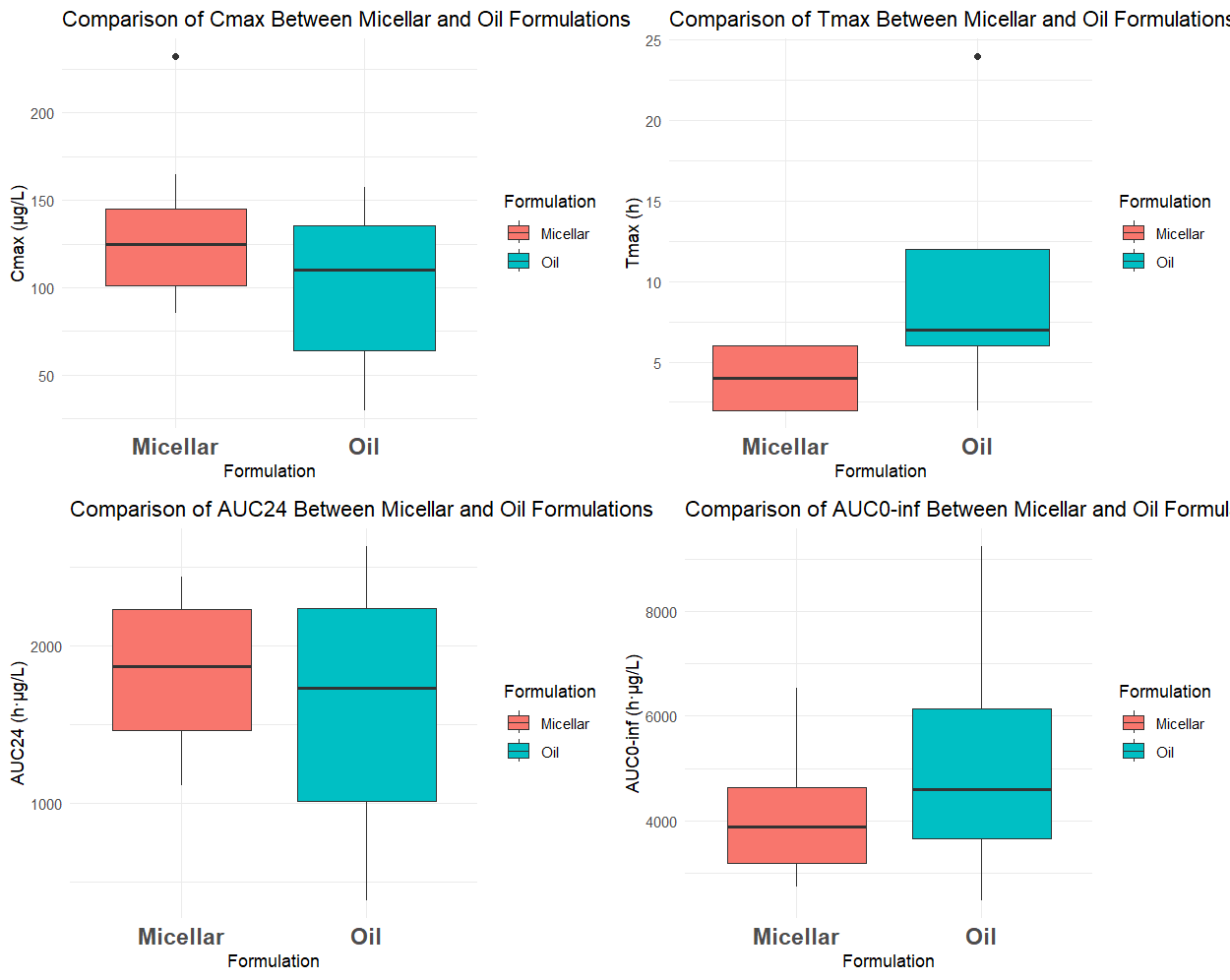


**Supplementary Figure 12**: Box plots for statistical comparisons between C_max_, T_max_, AUC_24_ and AUC_ss_ of CBG after simulated oral CBG administration for micellar (red) and oil (blue) formulations.


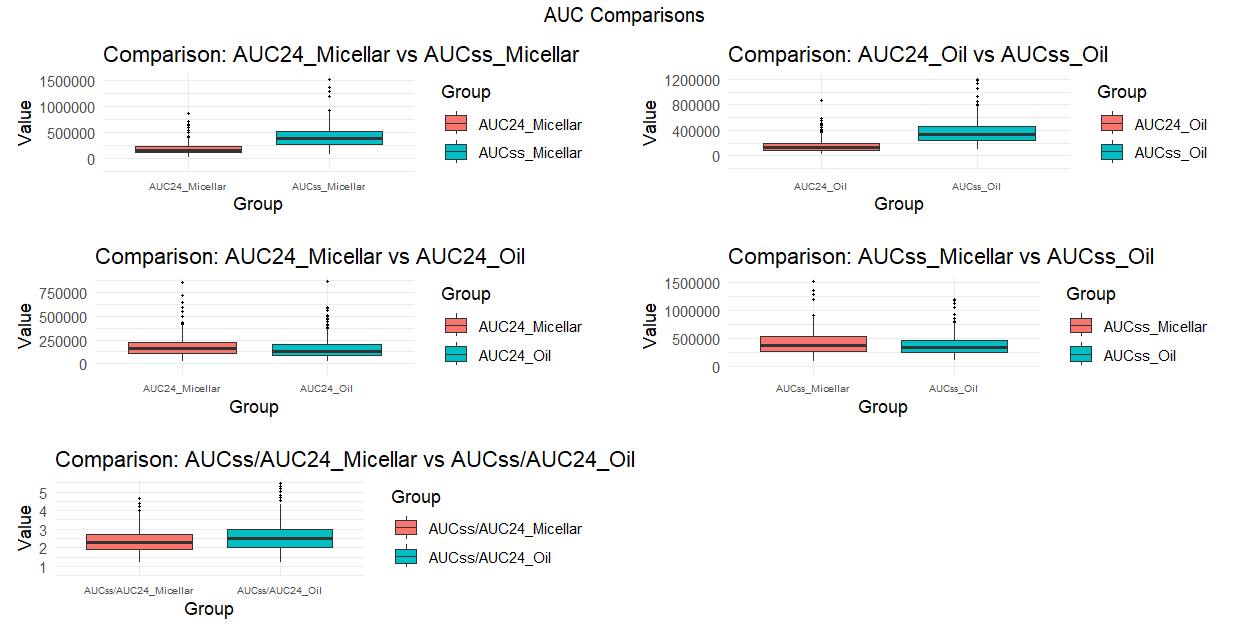


**Supplementary Figure** **13**: Box plots for statistical comparisons between AUC_24_ and AUC_ss_ for CBG-G after simulated oral CBG administration for micellar (red) and oil (blue) formulations.


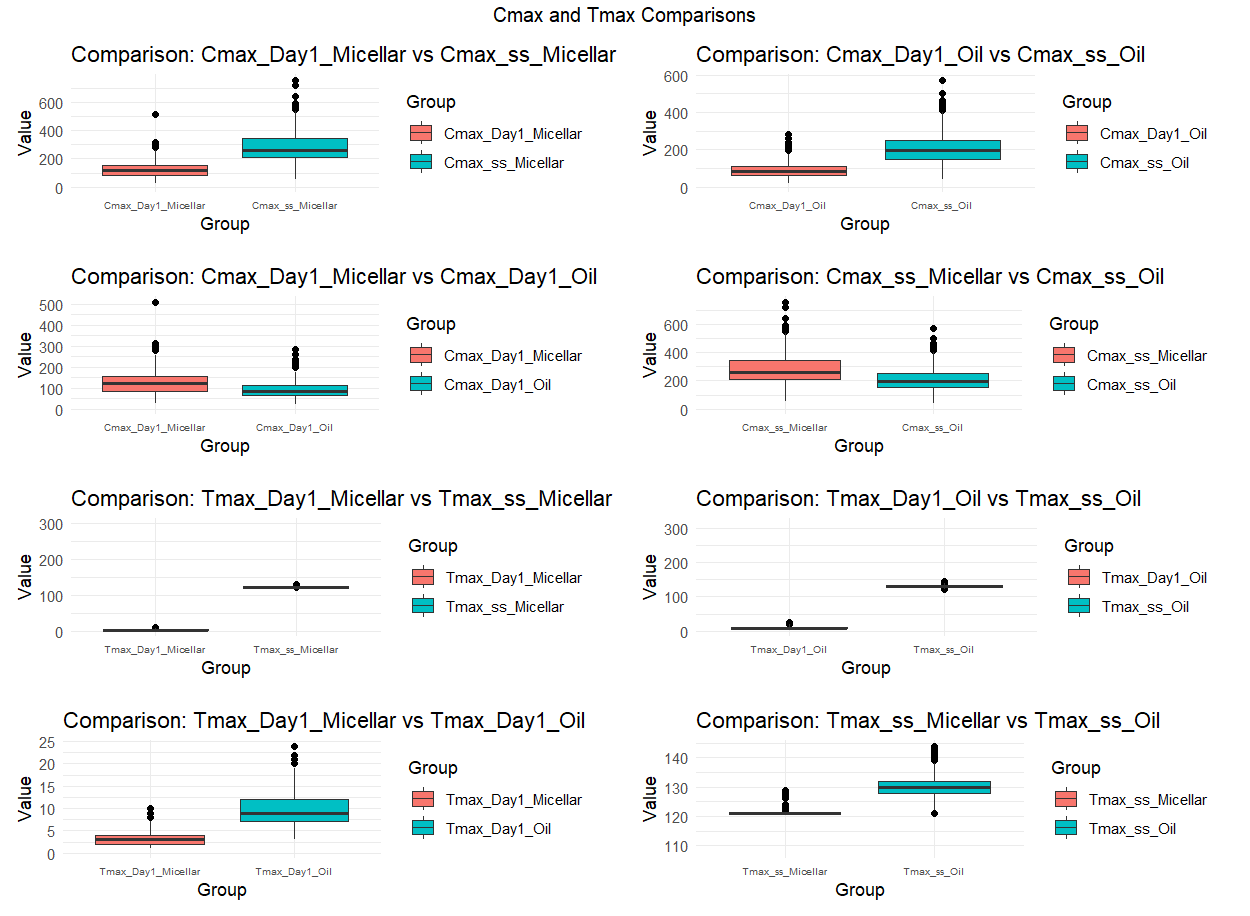


**Supplementary Figure 14.** Box plots for statistical comparisons between C_max_ and C_max-ss_, and T_max_ and T_max-ss_ for CBG after simulated oral CBG administration for micellar (red) and oil (blue) formulations.


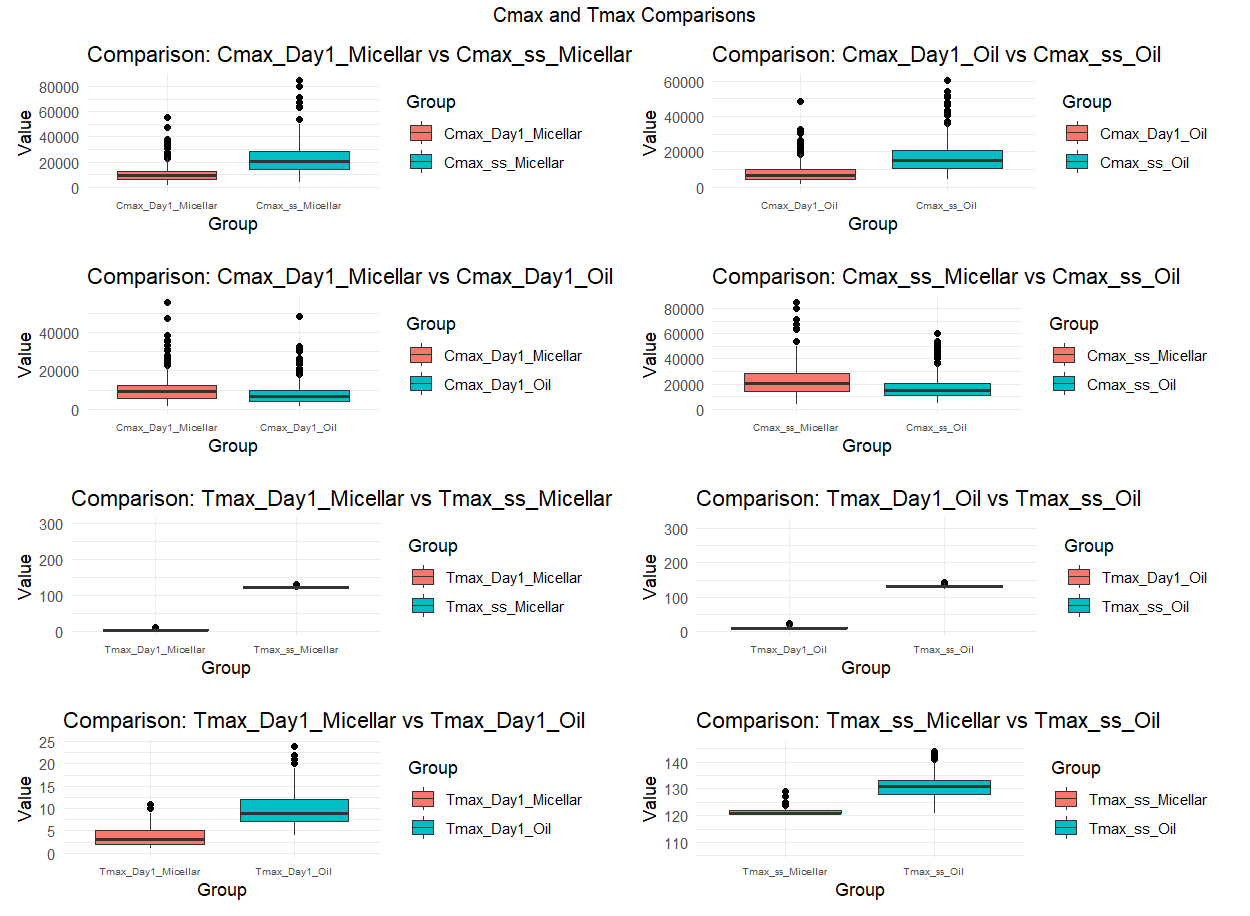


**Supplementary Figure 15.** Box plots for statistical comparisons between C_max_ and C_max-ss_, and T_max_ and T_max-ss_ for CBG-G after simulated oral CBG administration for micellar (red) and oil (blue) formulations.
